# Fermian guesstimation can boost the wisdom-of-the-inner-crowd

**DOI:** 10.1038/s41598-024-53639-3

**Published:** 2024-02-29

**Authors:** Tamara Gomilsek, Ulrich Hoffrage, Julian N. Marewski

**Affiliations:** Faculty of Business and Economics, Department of Organizational Behavior, University of Lausanne, Lausanne, Switzerland

**Keywords:** Psychology, Human behaviour

## Abstract

How can people’s ability to make accurate estimations be boosted? Psychological research on the wisdom-of-the-inner-crowd suggests that people’s judgments improve when they use a simple consider-the-opposite-strategy, dubbed—inspired by Enlightenment philosopher Hegel—dialectical-bootstrapping: A person generates a first estimate (thesis), then rejects it and generates another one (anti-thesis), and finally integrates both (synthesis). Yet, the wisdom-of-the-inner-crowd-phenomenon comes with controversy concerning its measurement, robustness, and moderators. We (1) introduce a novel class of strategies to elicit the wisdom-of-the-inner-crowd. These strategies root in physics, where Nobel-laureate Enrico Fermi used back-of-the-envelope guesstimation, for instance, when assessing the explosive yield of the first tested nuclear bomb. Fermian strategies prescribe decomposing an estimation problem into subtasks, solving the subtasks separately, and ultimately integrating those solutions into a final estimate. In an experiment using a new task-environment, we find (2) that a similarity-based Fermian-strategy boosts the wisdom-of-the-inner-crowd even more than consider-the-opposite does, (3) that the provision of a memory aid differentially affects those two strategies’ performance, and (4) that data trimming matters. Moreover, and for the first time, we document (5) overprecision in wisdom-of-the-inner-crowd estimations. Finally, we (6) replicate previous results, including that the collective intelligence of two persons still outperforms asking oneself twice.

## Introduction

Ever since Galton’s^1^ analysis, numerous replications revealed: (a) the average of many individuals’ independent estimates is typically closer to the truth than most of the separate estimates, and (b) its absolute error is lower than the average of the absolute errors of each of the estimates. This phenomenon became known as the *wisdom-of-crowds*^2–4^.

Yet, aggregation is also effective within an individual: the average of two estimates from the same individual tends to be better than the initial estimate of this individual—a phenomenon dubbed *wisdom-of-the-inner-crowd*^5–9^. While the wisdom-of-crowds-effect is well-established, there has been controversy about the wisdom-of-the-inner-crowd, specifically, its measurement^10^, robustness^11–13^, and moderators^14^. We contribute to the debate conceptually and methodologically, reporting an experiment featuring a novel estimation strategy, new dependent variables, new stimuli, and new analyses.

Our starting point is a foundational study by Herzog and Hertwig^6^ (henceforth, H&H). H&H asked participants in which year important historical events happened. Subsequently, participants were instructed to assume that their first estimate was off the mark, think of reasons why it might have been wrong, and ultimately provide a second—alternative—estimate. H&H found that the average of the first and second estimate of a given participant for a given event was, on average, closer to the truth than the first estimate alone. Moreover, their instruction led to averages closer to the truth than those of a control condition in which participants should provide a second estimate without following any strategy. Inspired by the philosopher Hegel, H&H subsumed their strategy of reconsidering under the label *dialectical-bootstrapping*: A process through which an initial thesis provokes an anti-thesis, calling for a synthesis. Consistent with H&H’s own terminology^6^ (p. 234; and with^15^, from whom H&H adopted the term), we will, henceforth, occasionally refer to dialectical-bootstrapping as “consider-the-opposite”. This is, however, not to suggest that there are no other strategies that can elicit dialectical estimates (see^6^, for an example see^16^; we will return to this point in the discussion).

### Goal 1: Benchmark Hegelian dialectical-bootstrapping against a strong competitor: Fermian guesstimation

Rather than only testing consider-the-opposite against a control condition, as H&H did, we also compared it to a representative of a novel class of competitors that we refer to as *Fermian*. Nuclear physicist and Nobel-laureate Enrico Fermi (1901–1954) is known for back-of-the-envelope guesstimation (e.g.,^17,18^). A Fermian-strategy decomposes a problem into several subtasks, tackles the subtasks separately, and ultimately integrates the solutions to those subtasks—a set of sub-estimates—into a final estimate. The advantage of such a procedure could be that overestimations in some subtasks and underestimations in others may cancel out each other, at least partially. The Fermian-strategy we focus on in this article is also known as a similarity-based strategy^19–22^; we introduce other members of this class in the discussion.

### Goal 2: Complete H&H’s design

H&H’s participants provided second estimates while seeing their first estimates, whereas those in the control condition were not shown their first estimates. It is therefore impossible to determine whether any difference between these two conditions should be attributed to the strategy (dialectical-bootstrapping versus no strategy) or to aiding participants’ memory of what they initially estimated. To disentangle these two factors, we orthogonally manipulated *strategy* (dialectical-bootstrapping, Fermian bootstrapping, control) and *memory-aid* (present or not), thereby completing H&H’s original design.

What could be the consequences of providing/withholding a memory-aid? Dialectical-bootstrapping, as instantiated by the consider-the-opposite strategy, requires participants to assume that their first estimate is off the mark. Aiding participants’ memory by showing them their first estimate helps them to venture a different estimate. Conversely, for the Fermian-strategy and the control condition, it might be better *not* to be reminded of the first estimate as this makes the second one more independent, whilst providing it would anchor the second estimate on the first. The independence of estimates is crucial for the wisdom-of-crowds-effect because independent estimates imply independent errors, which, in turn, increases the chance of errors canceling each other out^23,24^. The possible interaction of strategy and memory-aid therefore suggests that the confounding element was not a flaw in H&H’s design but served boosting the performance in each of the two conditions (see also^8^).

### Goal 3: Gauge the robustness of the wisdom-of-the-inner-crowd-effect in a new task-environment

Follow-up studies that tested variants of dialectical-bootstrapping strategies in domains other than historical-event estimation came to mixed conclusions: for some task-environments, variants of dialectical-bootstrapping were successful, for others not^12,13^. Aiding to assess the robustness of the wisdom-of-the-inner-crowd-effect generally and dialectical-bootstrapping specifically, our experiment used a novel task: estimating properties of animals.

### Goal 4: Gauge the effect of trimming

White and Antonakis^10^ re-analyzed H&H’s data with the same accuracy measure H&H had used alongside four new measures. When they used the same measure, they could not replicate the performance of H&H’s dialectical-bootstrapping-strategy exactly, and for the new measures, they even arrived at different conclusions regarding this strategy’s benefits (see^25^ for a reply). The discrepancy could be due to both author teams having used different procedures for measuring central tendencies. H&H used 20% trimmed averages to mitigate the effect of outliers, which essentially implies that only the middle 60% of a given participant-specific measure contributed to the arithmetic average, while the 20% highest and 20% lowest values determined the thresholds for these contributions^26^ (henceforth, *participant-trimming*). In contrast, White and Antonakis reported no such trimming, suggesting that their averages included all participants. To examine the robustness of the wisdom-of-the-inner-crowd-effect against trimming, we (1) used all data and computed averages across all participants, and (2) used all data but then calculated 20% trimmed averages of participant-specific metrics, like H&H. Moreover, we (3) used six different procedures to exclude outliers on an item level before computing any metrics. We consider such *item-trimming* to be a more appropriate way to generate metrics that are robust against outliers; however, we report methods and results for item-trimming under Supplementary Information and focus, below, on gauging the effect of the established participant-trimming procedure^26,27^ used by H&H.

### Goal 5: Analyze estimates with respect to over/-underprecision

In many judgment tasks, people are notoriously overconfident (or, for estimation tasks, overprecise; cf.^28^). Can different estimation strategies not only boost objective performance but also increase the accuracy of people’s confidence intervals around their estimations? Surprisingly, the combination of the wisdom-of-the-inner-crowd and over/-underprecision in estimation tasks has not yet been studied (but see^29^, who linked the wisdom-of-the-inner-crowd to over/-underconfidence in a pair-comparison task). We filled this gap by asking our participants to provide a lower and an upper limit for each estimate. The width of a given range is an indicator of how trustworthy participants believe their estimate to be and how much trust would be justified: If these ranges are too often too narrow, people are overprecise, and if they are too often too wide, they are underprecise.

Strategies designed to boost the wisdom-of-the-inner-crowd may reduce overprecision for two reasons: First, they may lead to improved estimates, thereby eventually justifying high trust. Second, they may increase the awareness that estimations are uncertain, which, in turn, may lead to wider ranges.

### Goal 6: Compare the wisdom-of-the-inner-crowd to the wisdom-of-crowds

What is better, asking yourself a second time or asking some random person? The typical finding is that the latter is more helpful than the former^5,6,30^. We propose a novel type of analysis for making such comparisons, based on frequencies rather than sizes of errors.

## Method

### Participants

We collected data from 292 Prolific “workers” who had ≥ 50 Prolific submissions, an approval rate ≥ 95% (following^31^). Toward the end of the experiment, in a follow-up question, we asked: “Can we trust your data for scientific research?” We excluded participants from the analysis who said no, which led to a final sample of 285 participants (154 females; *Mdn*_Age_ = 40 years, *SD*_Age_ = 12.5).

### Ethics declarations

The ethics commission of the Faculty of Business and Economics of the University of Lausanne (Commission interdisciplinaire d’éthique de la Faculté des HEC; CER-HEC) approved the study. This study was conducted according to relevant guidelines and regulations. Informed consent was obtained from all participants before the start of the study.

### Task, procedure, and design

The experiment had four parts: (1) first estimation, (2) second estimation, (3) pair-comparison task, and (4) follow-up questions, for which participants needed, in total and on an average, 62 min, and for which they earned a flat fee of GBP 9.20 (for details, see Supplementary Information). Here, we only report data from the first and second parts.

In the first part, participants provided estimates for 50 items (five properties for each of 10 species: *body-weight*, *brain-weight*, *lifespan*, *gestation-period*, *sleep-time* for *Arabian-Horse*, *Grey-Wolf*, *Plain-Zebra*, *Red-Fox*, *Asian-Elephant*, *Short-beaked-Dolphin*, *Bengal-Tiger*, *Angora-Goat*, *Chimpanzee*, *Giant-Panda*). The true value for a given item was determined as the average of two sources (see Supplementary Information). The item order was randomized for each participant. After having provided an estimate, participants were asked to specify a lower and an upper limit such that in 90% of the cases, the true values fall between these limits.

In the second part, we randomly assigned participants to one of the six conditions that resulted from completely crossing the factor strategy (Dialectical-bootstrapping, Fermian, Control) with the factor memory-aid (Aided, Unaided), labeled as *Aided-Dialectical*, *Aided-Fermian*, *Aided-Control*, *Unaided-Dialectical*, *Unaided-Fermian*, and *Unaided-Control*, respectively. Participants assigned to the Aided conditions had their first estimates displayed on the screen, whereas participants in the Unaided conditions did not receive such reminders. For each of the six conditions, participants were presented with the same 50 items, albeit in a new random order, and asked to provide a second estimate, again with a lower and an upper limit. Participants did not know beforehand that they would have to give a second estimate.

### Strategies

Before participants were asked “Now please provide your second estimate”, they received different instructions. Those assigned to the dialectical-bootstrapping-strategy of considering-the-opposite were instructed equivalently as in^6^ (see p. 234): “Step 1: Assume that your initial estimate was off the mark. Step 2: Think about a few possible reasons why that could be. Which assumptions and considerations could have been wrong? Step 3: What do these new considerations imply? Was your initial estimate rather too high or too low? Step 4: Based on this new perspective, make a second alternative estimate.” These instructions—and, correspondingly, those for the Fermian condition—were repeated for each item, with a specific reference to the target animal (see Supplementary Information).

Participants assigned to the Fermian-strategy received the following instructions: “Step 1: Think of other animals that are similar to the presented animal. Step 2: Of those animals, pick two that you have good knowledge about and come up with estimates for these two animals. Step 3: How do these two estimates compare to your initial estimate? Are they higher or lower? Step 4: Re-estimate the initial estimate by using these two estimates.”

Finally, participants in the control condition simply saw: “The second estimate can be identical to your first estimate, but it can also differ. For instance, if you in the meantime changed your mind.”

### Data-cleaning

We excluded all body-weight estimates of all participants because, due to a software error in the Aided-Fermian condition, the numbers these participants provided as their initial estimates were coupled with a wrong unit of measurement (had we excluded estimates of body-weight only in the Aided-Fermian condition, the results would have essentially been the same).

## Results

**Figure 1:**
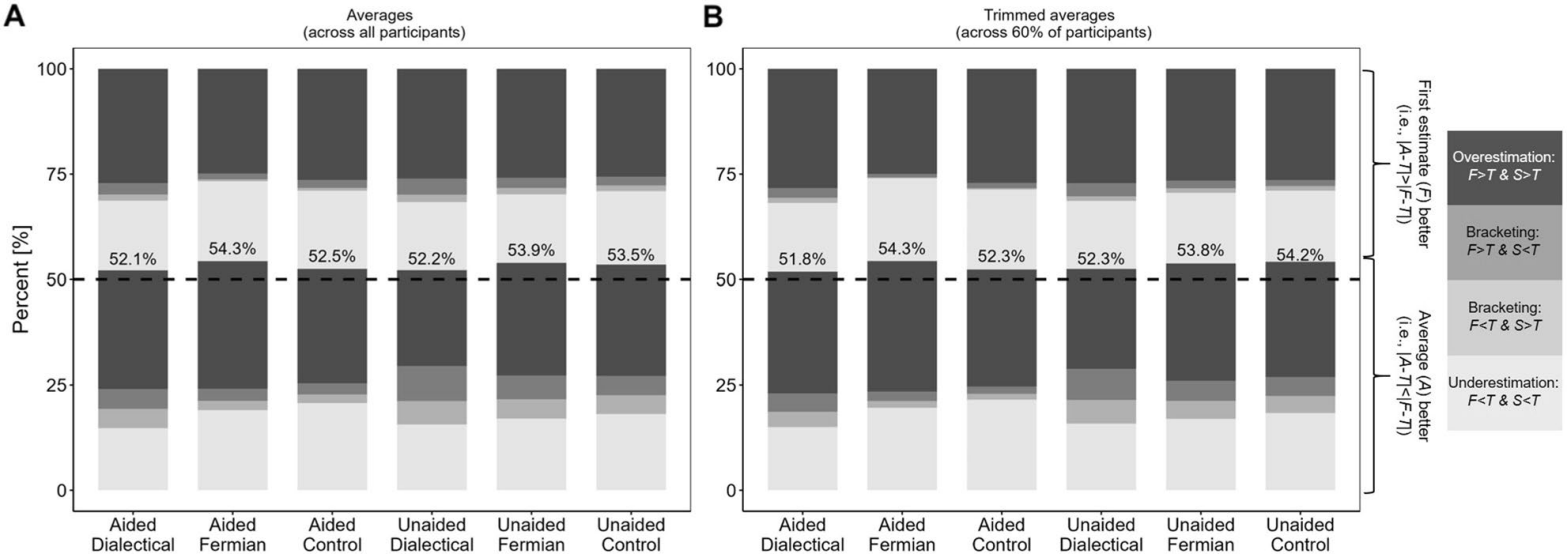
Constellations of First (*F*) and Second (*S*) estimates, and their Average (*A*), with respect to the True value (*T*). Bright bars: *F* < *T* & *S* < *T* (underestimation), bright grey: *F* < *T* & *S* > *T* (bracketing), dark grey: *F* > *T* & *S* < *T* (bracketing), and dark: *F* > *T* & *S* > *T* (overestimation). For the four bars at the bottom, below the dashed line, |*A* − *T*| < |*F* − *T*|, and for the four bars at the top: |*A* − *T*| > |*F* − *T*|. The black number in each condition is the sum of the four bars at the bottom. (**A**) displays averages of the participant-specific proportions, computed across all participants. (**B**) displays these averages computed across the 60% of participants that remain when using 20%-participant-trimming. Because the sum of the trimmed averages ranged between 95.0% and 97.7%, they were normalized within each condition to add up to 1.

**Figure 2:**
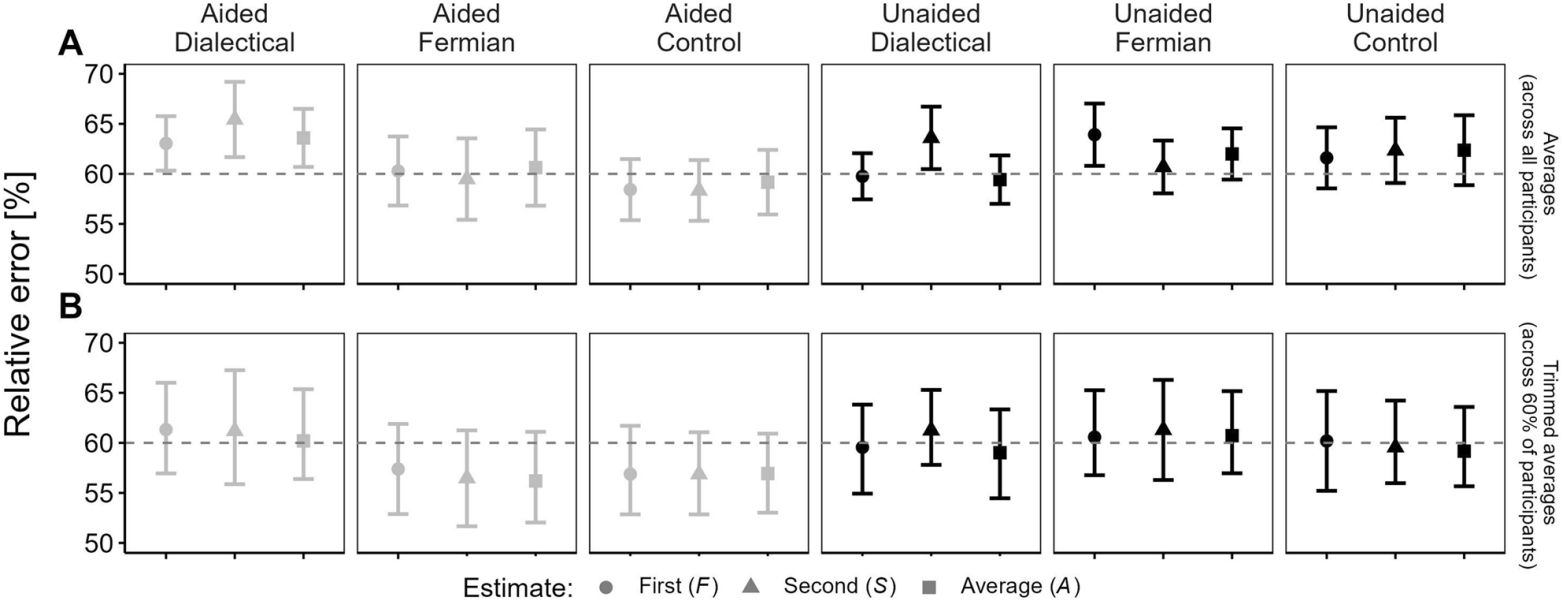
Relative error of First (*F*) and Second (*S*) estimates, and their Average (*A*) when compared to the True value (*T*). All relative errors are averaged (within a given condition; Eq. 2) across participant-specific medians [Eq. (1)]. The dotted horizontal lines aid comparisons of relative errors of *S* and *A* with the relative error of *F* within each condition. Comparisons of relative errors of *S* across conditions cannot be made because they had different reference points, namely the relative errors of *F* in those conditions, and these differences cannot be attributed to different treatments but only to sampling error. (**A**) displays averages of the proportions, computed across all participants, with vertical lines representing the ± 1 standard error of these averages, (**B**) displays 20%-trimmed averages of these proportions, with their 95%-bootstrap percentile intervals^27^.

**Figure 3:**
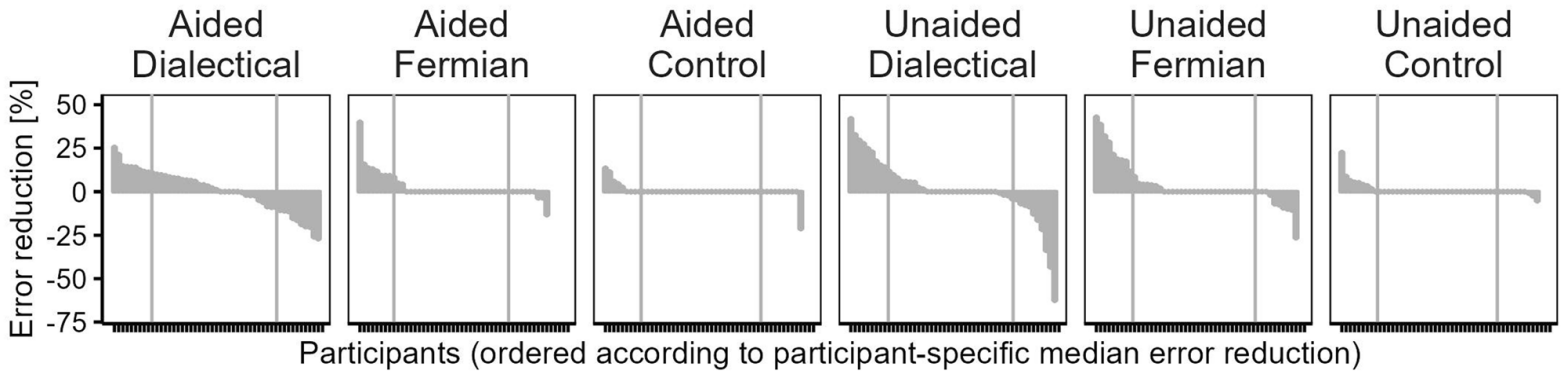
Distribution of participant-specific median error-reductions [Eq. (3)], separately for the six experimental conditions. The two vertical lines within each panel mark the 20 percentile and 80 percentile of each distribution.

**Figure 4:**
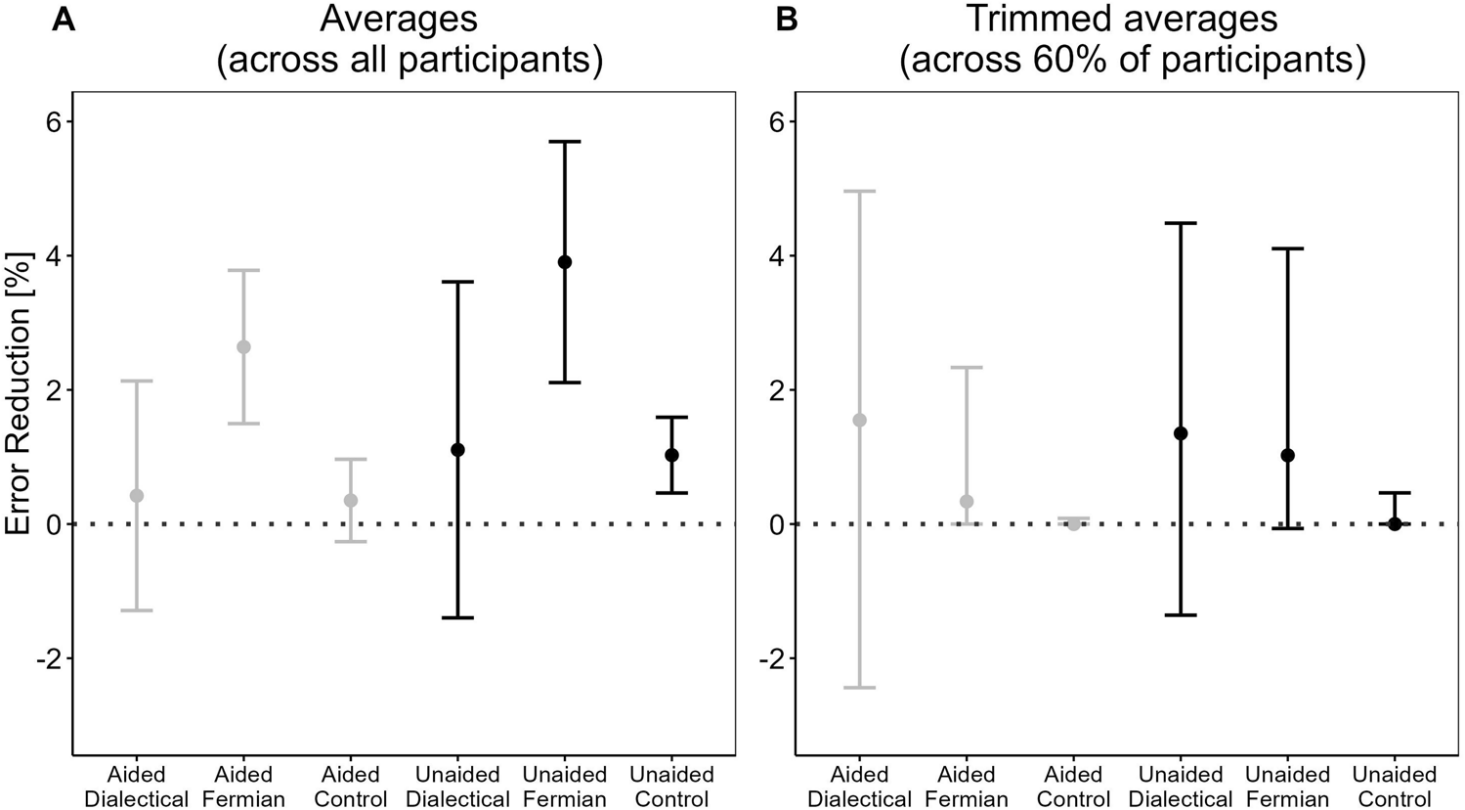
Averages [Eq. (4)] of participant-specific median error-reductions. (**A**) displays these averages computed across all participants, with vertical lines representing the  ± 1 standard error of these averages. (**B**) displays these averages computed across the 60% of the participants that remain when using 20%-trimming, with their 95%-bootstrap percentile intervals^27^.

We assessed the effects of our two independent variables (strategy and memory-aid) with four dependent variables (displayed at the y-axes of Figs. 1, 2, 3, 4) and start with these results. Subsequently, we inspect over-/underprecision and compare the wisdom-of-the-inner-crowd-effect to the wisdom-of-crowds-effect. Note that the animal properties had different scales (weight and time), but all dependent variables were constructed such that the data could be collapsed across all properties.

### Percentages of various constellations between first, second, and averaged estimates

Our first steps were to build categories depending on the relationship between the first (*F*) and the second estimate (*S*) for each item of each participant, to their arithmetic average *A* (with *A* = (*F* + *S*)/2), and to the true value (*T*). The four bars at the bottom of the piles in Fig. 1 denote the percentages of items—first determined within participants, and subsequently averaged across participants—for which the absolute error of *A* (i.e., |*A* − *T*|) was smaller than the absolute error of *F* (i.e., |*F* − *T*|), and the four bars at the top denote those percentages of items for which |*A*-*T*| > |*F* − *T*|. Items for which |*A* − *T*| = |*F* − *T*| were equally split between the corresponding bottom and top bars. This happened in 3.9%, 38.4%, 52.8%, 14.8%, 21.0%, and 24.0% of the conditions, from left to right in Fig. 1A, respectively, mostly when *F* and *S* were identical. (These percentages also include the other possibility, namely |*A* − *T*| = |*F* − *T*| and *F* ≠ *S*, but such cases occurred in only 0.2% across all conditions.)

Providing *S* and subsequently averaging *F* and *S* more often led to an improvement over considering *F* alone: In each of the six conditions, the sum of the four lowest categories—this sum is displayed in Fig. 1 above the dotted line—exceeded 50%. The difference between this number to 50% can be considered as one measure of the wisdom-of-the-inner-crowd-effect, with Δ = 2.1 (*SD* = 10.1), 4.3 (*SD* = 8.6), 2.5 (*SD* = 6.8), 2.2 (*SD* = 11.0), 3.9 (*SD* = 10.9), and 3.5 (*SD* = 6.8), and Cohen’s *d* = 0.212, 0.503, 0.365, 0.20, 0.357, and 0.519, respectively (*p* = 0.072, < 0.001, 0.007, 0.084, 0.008, and < 0.001). The effect of strategy was F(2,279) = 1.07, *p* = 0.345 and the effect of memory-aid was F(1,279) = 0.037, *p* = 0.847 (for the interaction, F(2, 279) = 0.147, *p* = 0.863). Given that memory-aid had no effect and did not interact with strategy, we pooled across memory-aid-conditions when we determined the following contrasts: Dialectical-bootstrapping vs. Control, *t*(167) = 0.659, *d* = 0.094, *p* = 0.511; Fermian vs. Control, *t*(163.3) = − 0.896, *d* = − 0.131, *p* = 0.372; and Dialectical-bootstrapping vs. Fermian, *t*(189) = 1.312, *d* = 0.191, *p* = 0.189.(For the contrasts Dialectical-bootstrapping vs. Control and Fermian vs. Control we used Welch’s t-test because the variances were not homogenous.)

H&H explained the wisdom-of-the-inner-crowd-effect in terms of *bracketing* (adopting this idea from^32^, who had used it previously for the wisdom-of-crowds-effect; see also^3,33^. Bracketing of *T* occurs when a participant’s two estimates fall on the opposite side of the *T*. Whereas the white and black bars in Fig. 1 decode constellations for which both estimates fall on the same side (white: *F* < *T* & *S* < *T*; black: *F* > *T* & *S* > *T*), the two grey bars decode cases of bracketing (light grey: *F* < *T* & *S* > *T* and dark grey: *F* > *T* & *S* < *T*).

Bracketing more often led to an improvement of *A* over *F* than not. The percentage points of the two grey bars below the dotted line in Fig. 1A (for which |*A* − *T*| < |*F* − *T*|), henceforth *helpful bracketing*, summed up to 9.3, 5.1, 4.7, 13.9, 10.2, and 9.0, for the six conditions, respectively. Those above the dotted line, henceforth *unhelpful bracketing*, were 4.1, 1.8, 2.5, 5.6, 3.9, and 3.4—here, *S* was so far on the other side of *T* that it would have been better to ignore *A* and to take *F* alone. The difference between the percentage points of helpful and unhelpful bracketing indicates how bracketing contributed to the wisdom-of-the-inner-crowd-effect: 5.1, 3.2, 2.1, 8.3, 6.3, and 5.6 (5.1 across all items and conditions). Note that these percentage points were, on average, even higher than the wisdom-of-the-inner-crowd-effects reported above. A closer examination of the bracketing cases computed across all experimental conditions revealed that 70.6% of them were helpful, whereas the other 29.4% were unhelpful.

Not aiding participants’ memory should lead to less anchoring on *F* and, hence, more independent *S*. Indeed, there were more cases of bracketing for the unaided conditions than in the respective aided conditions. Finally, a comparison of Fig. 1A and B reveals that participant-trimming did not affect the results substantially.

### Accuracy of estimates

In addition to the percentages mentioned above, which ignore sizes of estimation errors and their reductions, we also used measures that take these sizes (and reductions) into account. In a first step, we determined the relative error of a given participant’s *F* for a given item as the relative size of the absolute difference between *F* and *T* compared to *T*. In a second step, we determined the median of these relative errors per participant *p* across all *i* = 40 items (without body-weight estimates and without trimming on the item level; Eq. 1). In a third step, we averaged the participant-specific medians across participants of a certain condition *c* [Eq. (2)]. The same measure was also computed for *S *(with *S* replacing *F* in Eq. 1) and for *A* (with* A* replacing *F*).

1 $${Relative \, error}_{p}= {Mdn}_{i=1}^{i=40}\left(\left(\frac{\left|{F}_{i}- {T}_{i}\right| }{{T}_{i}}\right)*100\right)$$

2 $${Relative \, error}_{c}= Average ({Relative \, error}_{p})$$

Figure 2A shows that, in both memory-aid-conditions, the Fermian-strategy helped participants to reduce their relative error of *S* compared to that of *F*, albeit only slightly. In contrast, in both dialectical-bootstrapping-conditions, the relative error of *S* even increased (again, only slightly). In both control conditions, aided and unaided, the relative errors of *F* and* S* were roughly the same. Figure 2B shows that the superiority of the Fermian-strategy over dialectical-bootstrapping could no longer be observed when trimmed averages of participant-specific relative errors of *F* and *S* were used.

### Error-reduction

The error-reduction captures how the absolute errors of *A* compare to those of *F*. Like^6^ (p. 234) and^10^ (p. 115), we computed, first, Eq. (3) within each participant *p* (across *i* = 40 items); second, we averaged these participant-specific medians across all participants of a certain condition *c* [Eq. (4)].

3 $$Error \text{-} reduction_{p}= { Mdn}_{i=1}^{i=40}\left(\frac{\left|{F}_{i} - {T}_{i}\right|-\left|{A}_{i}-{T}_{i}\right|}{\left|{F}_{i} - {T}_{i}\right|}\right)$$

4 $$Error \text{-} reduction_{c} = Average (Error \text{-} reduction_{p})$$

Figure 3 displays the distribution and magnitudes of participant-specific median error-reductions [Eq. (3)], Table 1 displays the corresponding percentages of participants for whom the median error-reduction was positive (%pos; ‘winners’), zero (%zero), or negative (%neg; ‘losers’), and Fig. 4 displays the averages of participant-specific median error-reductions [Eq. (4)].

**Table 1:** Categories of participants, defined by their median error-reduction.

Condition	N (participants)	Participants with positive median error-reduction (%pos)	Participants with median error-reduction of zero (%zero)	Participants with negative median error-reduction (%neg)	Difference %pos − %neg (*D*)
Aided-Dialectical	49	51.0	10.2	38.8	12.2
Aided-Fermian	45	24.4	68.9	6.7	17.8
Aided-Control	47	12.8	85.1	2.1	10.6
Unaided-Dialectical	49	36.7	34.7	28.6	8.2
Unaided-Fermian	48	33.3	52.1	14.6	18.8
Unaided-Control	47	17.0	76.6	6.4	10.6
Data pooled across the aided and unaided conditions					
Dialectical	98	43.9	22.4	33.7	10.2
Fermian	93	29.0	60.2	10.8	18.3
Control	94	14.9	80.6	4.3	10.6

Percentages of participants per category are based on all participants of a given condition—the same participants that contributed to the averages displayed in Fig. 4A.

In the Aided-Dialectical-condition, in which participants were asked to provide *S* such that it differs from *F*, and in which they were shown *F*, very few (10.2%) had a median error-reduction of zero (Fig. 3, Table 1). In the Aided-Fermian and the Aided-Control-condition, in which *S* did *not* need to differ from *F*, the picture reversed: here, even the majority (68.9% and 85.1%, respectively) had a median error-reduction of zero. In the Unaided-Dialectical-condition—in which *F* was not shown, thereby providing no help what *S* should differ from—the percentage of participants with a median error-reduction of zero increased substantially (to 34.7%) compared to the Aided-Dialectical-condition, whereas for Unaided-Fermian and Unaided-Control, this percentage barely differed from their aided counterparts.

Table 1’s right-most column reports the difference, *D*, computed by subtracting %neg from %pos. In each of the six conditions, *D* > 0, with *D* being largest in the Unaided-Fermian-condition. The superiority of the Fermian-strategy also emerged when *D* was determined after having pooled the aided and unaided conditions.

Figure 4A displays the averages [Eq. (4)] of the participant-specific median error-reductions from Fig. 3. The reduction was largest in the two Fermian conditions, Aided and Unaided (2.6% and 3.9%, and also significantly different from zero, *p* = 0.013 and *p* = 0.017, with *d* = 0.34 and *d* = 0.31, respectively). For the direct comparisons of the Fermian-strategy with the dialectical-bootstrapping-strategy (across both memory-aid conditions), we computed *p* = 0.17, *d* = 0.2 (and for Fermian against control, *p* = 0.013, *d* = 0.33; Dialectical against control, *p* = 0.481, *d* = 0.007). For the other main factor, memory-aid (Aided vs. Unaided; across all strategy conditions), we observed *p* = 0.48 and *d* = 0.08.

When the central tendency of participant-specific median error-reductions was computed with trimmed averages (Fig. 4B), the pattern reversed: Now error-reduction was largest in the two Dialectical conditions (1.55% and 1.35%, with *p* = 0.086 and *p* = 0.026 against a baseline of zero, as well as *d* = 0.251 and *d* = 0.363, for Aided and Unaided, respectively). For the Fermian conditions, it dropped to 0.34% and 1.02% (with *p* = 0.081 and *p* = 0.006, and *d* = 0.277 and *d* = 0.486, for Aided and Unaided, respectively). For the direct comparisons of the Fermian-strategy with the dialectical-bootstrapping-strategy (across both memory-aid conditions), we computed *p* = 0.274 and *d* = 0.196. For Fermian against control, *p* = 0.002 and *d* = 0.563, and Dialectical against control, *p* = 0.014 and *d* = 0.40 For the main factor, memory-aid (Aided vs. Unaided), across all strategy conditions, we observed *p* = 0.757 and *d* = 0.047.

Comparing Fig. 4A and B reveals that the superiority of Fermian-strategies with respect to error-reduction disappeared when using trimmed averages. How can this be explained? Recall that the calculation of a trimmed average uses only the 60% of participants in the middle of a given distribution in Fig. 3. Therefore, the difference between the pattern for untrimmed averages (largest error-reduction for Fermian, Fig. 4A) and trimmed averages (largest reduction for Dialectical, Fig. 4B) can best be understood by examining those 40% of participants in the tails that have been excluded from the computation. For both dialectical conditions (Aided and Unaided), each of the tails contained only participants with either positive (left tails in Fig. 3) or negative (right tails) error reductions, that is, each of the corresponding numbers in Table 1 exceeded 20%. Moreover, the area in the left tails was roughly the same as the area in the right tails, both for Aided and Unaided (Fig. 3). In contrast, for the Fermian conditions, only the left tails were completely filled with participants who had positive median error-reductions, whereas the right tails also contained many with a median error reduction of zero (Table 1). Moreover, the area in the left tails exceeded, by far, the area in the right tails, both for Aided and Unaided (Fig. 3). Due to these differences with respect to the tails of the distributions, trimming affected the two strategies differentially. Bluntly speaking, trimming had relatively little effect in the Dialectical conditions, because the ‘winners’ (in the left tails) and the ‘losers’ (in the right tails) that were left behind when computing the trimmed average almost cancelled out each other. In contrast, the Fermian strategies had more ‘winners’ than ‘losers’, and exactly this competitive advantage could no longer play a role when computing the trimmed averages with truncated tails.

A corollary of the differential effect of trimming on strategies discussed above can be seen in Table 2 which reports recalculated %pos, %zero, %neg, and *D* for only those participants between the 20 and 80 percentiles shown in Fig. 3. In particular the comparison of *D* in Tables 1 and 2 reflects that—and why—Dialectical-bootstrapping appears in a better light when trimmed averages are used.

**Table 2:** Categories of participant, defined by their median error-reduction.

Condition	N (participants)	Participants with positive median error-reduction (%pos)	Participants with median error-reduction of zero (%zero)	Participants with negative median error-reduction (%neg)	Difference %pos − %neg (*D*)
Aided-Dialectical	31	38.7	25.8	35.5	3.2
Aided-Fermian	27	0	100	0	0
Aided-Control	29	0	100	0	0
Unaided-Dialectical	31	13.3	80	6.7	6.7
Unaided-Fermian	30	0	93.3	6.7	-6.7
Unaided-Control	29	0	100	0	0
Data pooled across the Aided and Unaided conditions					
Dialectical	62	26.2	52.5	21.3	4.9
Fermian	57	0	96.5	3.5	3.5
Control	58	0	100	0	0

Percentages of participants per category are based on only those participants that were included in the calculation of the trimmed averages displayed in Fig. 4B.

### Over-/underprecision

**Figure 5:**
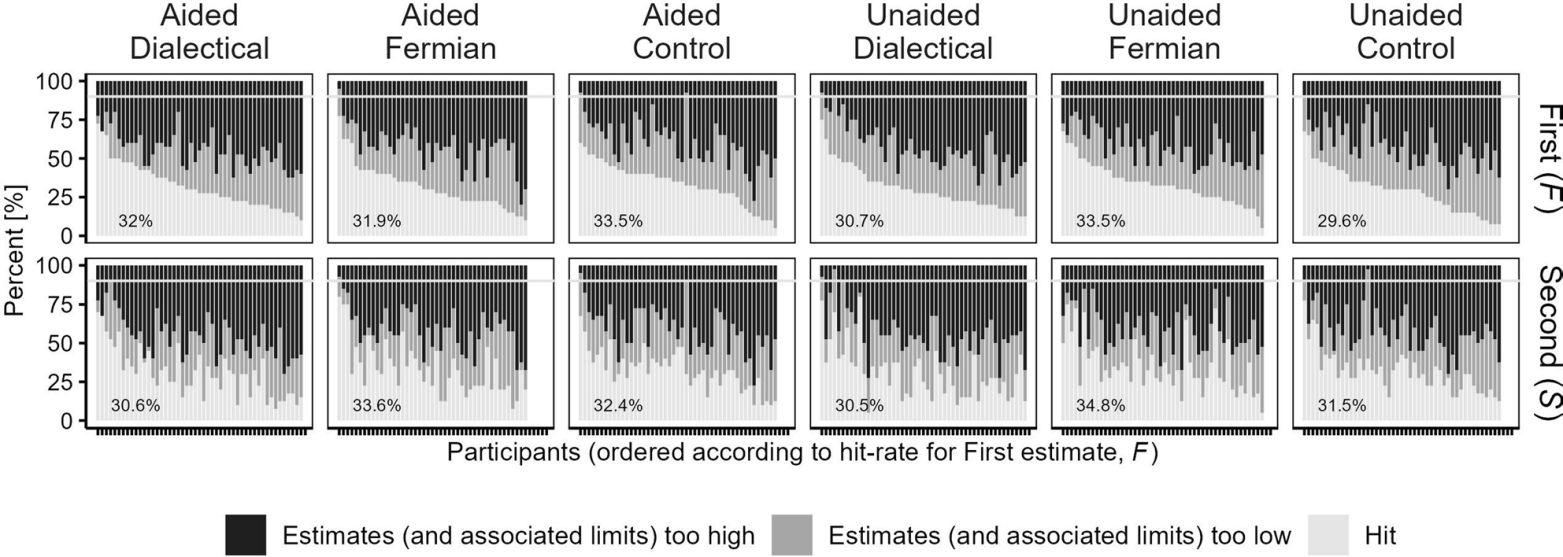
The percentage of items for which the True value (*T*) fell between the specified limits (hit), estimates (and associate limits) were too high, or estimates (and associate limits) were too low. In each panel, the number denotes the average hit-rate.

We defined over-/underprecision as the percentage of true values that fell between the limits specified by participants (henceforth, the percentage of hits). Given that participants were instructed to set the lower and upper limits such that 90% of the true values fall between them, a hit-rate of 90% would indicate precisely chosen confidence intervals, and a hit-rate below (above) would indicate that ranges were too narrow (wide). The distribution of participants’ hit-rates revealed gross overprecision (ranges too narrow), with almost no difference between conditions (Fig. 5): Instead of being 90% (marked as a white line), the averages of participants’ hit-rates (each computed within a given condition) spread, across conditions, from 29.6 to 33.5% for the first estimate and from 30.5 to 34.8% for the second estimate. For most of the remaining items, the estimates (and their two associated limits) were too high, consistent with the fact that the scale was open-ended for high values but was restricted at the lower end.

Did overprecision decrease for *S*? Notably the consider-the-opposite strategy, but maybe also the Fermian-strategy (and arguably also the control condition) could have raised doubts in *F*, which may have led to wider ranges for *S* and, hence, more hits and less overprecision. Results were mixed (Fig. 5): For the two Fermian-conditions, overprecision indeed decreased, albeit only slightly (by 1.7 and 0.9 percentage points for Aided and Unaided, respectively); for the two dialectical-bootstrapping-conditions, it even increased (but again, only slightly: by 1.4 and 0.2 percentage points for Aided and Unaided), and for the control condition, it increased when a memory-aid was provided (by 0.9 percentage points), and it decreased when not (1.9 percentage points). Across all conditions, for 43.2% of the items, the ranges around *F* were larger, for 39.6% those around *S* were larger, and for 17.2%, they were equally large, with no substantial differences between conditions.

When we asked participants, after the second part, “how many of the true values do you think are actually inside your ranges?”, the average response, across all participants, was 61.7% (*SD* = 22.4) indicating that participants believed their ranges were too narrow, but still underestimated the extend to which this was the case.

### Wisdom-of-the-inner-crowd vs. wisdom-of-crowds

We compared the beneficial effect of generating a second estimate to that of asking another individual. Specifically, for each item of a given participant, we compared |*A* − *T*| to |*A*_*Fi*_ − *T*|, with *A*_*Fi*_ being the average of this participant’s *F* with *F* of another participant (*i*), resulting in three possible outcomes: *A* could be closer to* T* (in Table 3 referred to as “better to ask oneself a second time”), *A*_*Fi*_ could be closer to *T* (“better to ask someone else”), and both could produce identical errors. Performing these comparisons for a given participant and a given item with the estimates of all other participants (of the same experimental condition) yielded three percentages. We computed a fourth percentage by subtracting the percentage of “better to ask oneself a second time” from the percentage of “better to ask someone else”. This difference can be seen as a measure for the superiority of asking another individual over asking oneself a second time. Each of these four percentages, obtained for a given item of a given participant, was then averaged across all items of this participant. Ultimately, we computed the averages (and their standard errors) across all participants of the same condition.

**Table 3:** Robustness of the superiority of asking someone else over asking oneself a second time.

Condition	Outcome of comparison							
	Better to ask oneself a second time		Identical error		Better to ask someone else		Superiority of asking someone else	
	*M* [%]	*SE* [%]	*M* [%]	*SE* [%]	*M* [%]	*SE* [%]	*M* [%]	*SE* [%]
Aided-Dialectical	42.8	1.3	7.3	0.3	50.0	1.4	7.3	2.7
Aided-Fermian	43.3	1.4	7.6	0.3	49.0	1.6	5.7	3.0
Aided-Control	41.2	1.3	8.1	0.3	50.7	1.3	9.4	2.7
Unaided-Dialectical	42.3	1.2	7.1	0.3	50.5	1.3	8.2	2.5
Unaided-Fermian	43.6	1.7	7.4	0.4	49.0	1.8	5.3	3.5
Unaided-Control	43.0	1.6	6.7	0.4	50.3	1.6	7.3	3.2

As shown in Table 3, in each of the six conditions, participants would more often benefit if they asked another participant for their first estimate than if they asked themselves a second time *(M* difference = 8.3%, *SE* = 0.8%). The superiority of asking someone else was smallest for the two Fermian-conditions, which is consistent with the observation that Fermian-participants had the highest error reductions (from *F* to *S*, Fig. 4A): Those who benefited most from asking themselves a second time could, compared to the other conditions, gain less by asking others.

## Discussion

Hegelian dialectical-bootstrapping, as implemented by H&H’s consider-the-opposite instruction, prescribes assuming *F* is off the mark (Box 1, Step 1), searching for reasons confirming that *F* is erroneous (Box 1, Step 2), and using those reasons to construct *S* (Box 1, Steps 3&4). These principles exhibit five weaknesses, §1–§5, that are listed on the left-hand side of the text that follows below.

Fermian estimation prescribes decomposing a problem in *n*_*Fermian*_ ≥ 2 subtasks and aggregating the subtask solutions to generate *S*. Fermian-strategies avoid the weaknesses of the dialectical strategy of considering-the-opposite and have additional strengths, §6–§11, listed on the right-hand side of the following text (see also Supplementary Information). To facilitate direct comparison, the strengths of the Fermian-strategy tested here are organized to mirror the weaknesses of dialectical-bootstrapping, yet we recommend first reading column-wise, and only then line-wise to venture to comparisons between the strategies.

**Table:** 

Dialectical-bootstrapping: Consider-the-opposite	Fermian estimation: Decompose and aggregate
(§1) Consider-the-opposite builds on—and hence requires—*F* (simply because “opposite” implies “opposite of something”).	(§6) *F* is, technically, not part of Fermian-strategies. Indeed, Fermians do not need *F* to generate wise estimates (§10–11)
(§2) If, in reality, *F* was *not* off the mark, the reasons (e.g., data and other evidence) suggesting that *F* is off the mark are likely misleading. Then *S* risks being built on misleading assumptions.	(§7) Unlike dialectical-bootstrapping (§2), Fermian estimation does not require using evidence disconfirming *F* to construct *S*. Hence, evidence feeding into Fermian *S* is, algorithmically, independent of *F.*
(§3) When generating *S*, dialectical-bootstrappers cannot treat *F* as being correct even if they had strong evidence to believe so.	(§8) Unlike dialectical-bootstrappers (§3), Fermians can generate *S* identical to *F.*
(§4) Estimation errors are hoped to cancel out by averaging *F* and *S*, but this can happen only if *S* is either closer to *T* (*F* < *S* < *T* or *F* > *S* > *T*), or if *F* and *S* bracket *T* in a helpful way (|*A − T*| < |*F − T*|).	(§9﻿) A naïve *F* is not required (§6). If it is nevertheless given, estimation errors in naive *F* and Fermian *S* can cancel each other out (§4). Irrespective of this, errors within a Fermian *S* can also cancel each other out (§10).
(§5) *A* is computed across two estimates, *F* and *S*, where *S* is only a single number that results from rejecting *F*. We write ‘only’, because *S* does not result from aggregating several numbers as in Fermian bootstrapping (see §10–11).	(§10) To preserve parallelism with the dialectical-bootstrapping-condition, *A* in our Fermian-condition is nevertheless computed across *F* and *S*, but unlike for dialectical-bootstrappers (§5), an *S* of Fermians results from aggregating *n*_*Fermian*_ subtask estimates (with *n*_*Fermian*_ ≥ 2 in general, and *n*_*Fermian*_ = 2 in our study). Hence, the total numbers of estimates, *N*, that enter the calculation of *A* differ, with *N*_*Fermian*_ > *N*_*Dialectical-bootstrapping.*_
	(§11) The size of *n*_*Fermian*_ is unlimited. As long as the errors in *n*_*Fermian*_ subtask estimates are not strongly correlated, the larger *n*_*Fermian*_, the more accurate a Fermian *S* will be.

Consistent with this analysis (§1–10), we found: For the majority of Fermians, the median of error-reductions was zero, and among the other Fermians, most benefited from generating different *S* (more ‘winners’ than ‘losers’; Fig. 3, Table 1), also echoed by Fermian’s average error-reduction (Fig. 4A). In contrast, pushing for *S* that deviate from *F*, dialectical-bootstrapping resulted for almost all participants in median error-reductions different from zero (Table 1), and that was, on average, not very helpful (Fig. 4A). Overall, the average error-reduction was largest in the Fermian-conditions, whereas for the dialectical-bootstrapping-conditions it was virtually indistinguishable from the control conditions (Fig. 4A).

However, this pattern of results reversed when using participant-trimmed averages, which barely changed the effect for Dialectical-bootstrapping, but handicapped the Fermian-conditions by eliminating, relatively speaking, more ‘winners’ than ‘losers’ from the effect size measure (Fig. 4B). Hence, the participant-trimming procedure used by H&H can dramatically change the patterns of results. We did not observe such a change when trimming data on the item level. The differential effect of the trimming procedure could possibly be explained, for instance, because participant-trimming entails ranking and building subsets of participants directly based on the metric of interest (e.g., error-reduction), whereas item-trimming is based on *F* and *S* (see Supplementary Information).

Importantly, for the Fermian-conditions *S* actually improved compared to *F*, whereas for the dialectical-bootstrapping-conditions *S* got worse (Fig. 2A), and indeed, the wisdom-of-the-inner-crowd-effect, Δ, was the largest in the Fermian-conditions (Fig. 1A), and that despite helpful bracketing occurring most often—and contributing most often to Δ—in the dialectical-bootstrapping-conditions. Finally, room for benefitting from others’ *F* was smallest in the Fermian-conditions (Table 3).

**Table:** 

Dialectical-bootstrapping: Consider-the-opposite	Fermian estimation: Decompose and aggregate
(§12) Without *F* (thesis) dialectical-bootstrappers cannot construct *S* (anti-thesis) (§1).	(§13) While Fermians do not need *F* (§6), if* A* is nevertheless computed across *F* and Fermian *S* (§10), then *not* being reminded of *F* makes errors in Fermian *S* more independent from errors in *F*, likely boosting *A*’s accuracy.

Consistent with §12–13, *D* was larger in the Aided-Dialectical condition than in the Unaided-Dialectical-condition; conversely, Fermians’ *D* was larger in the Unaided than in the Aided condition (Table 1), suggesting that the strategies’ predilected areas of applicability may differ (cf.^34^). Yet, if a person could choose (a) whether memory is aided and (b) what strategy to use, the best advice would be to prefer the combination Unaided-Fermian over Aided-Dialectical (Figs. 1A, 2A, 3, 4A, Table 1).

**Figure 6:**
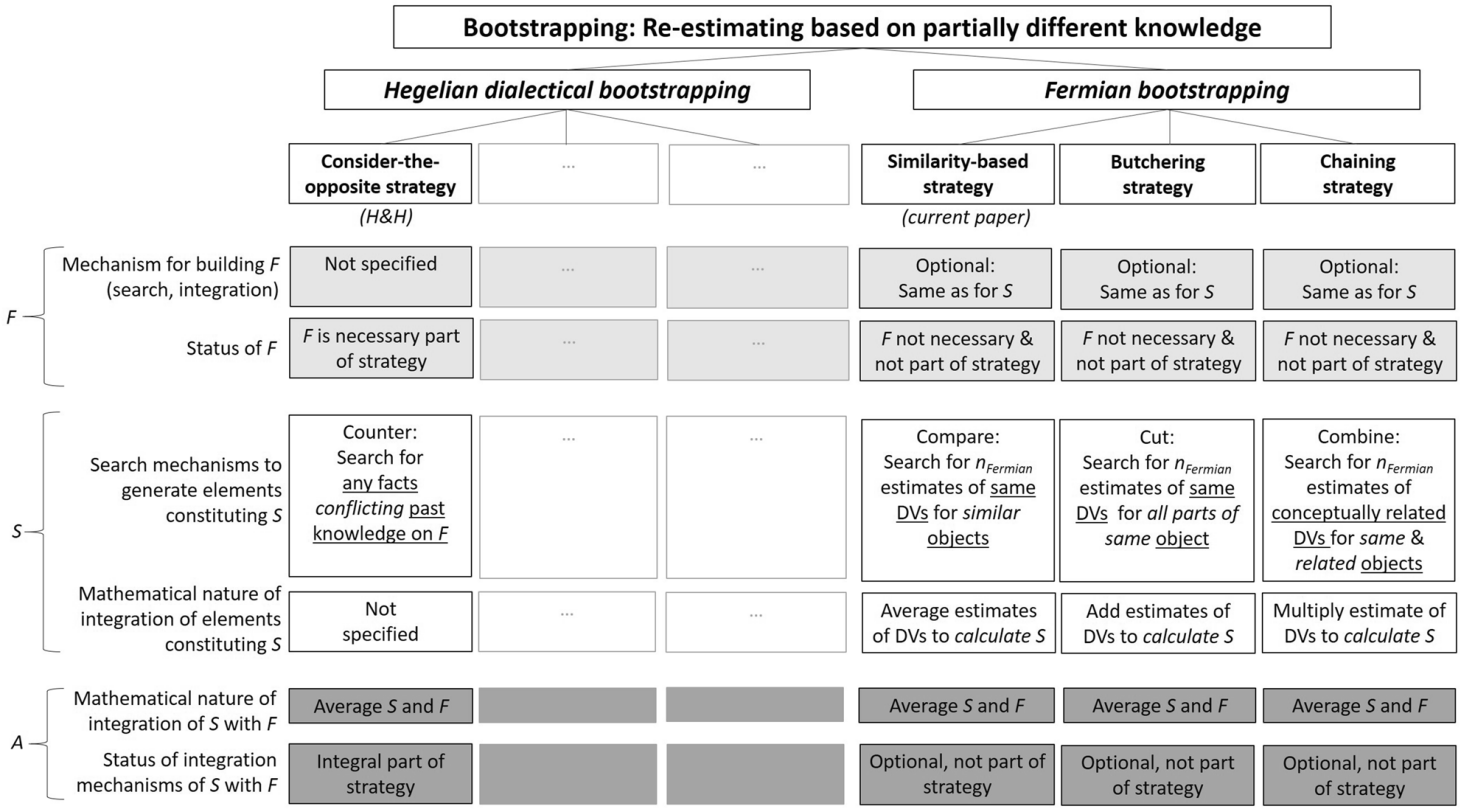
Towards a framework for understanding bootstrapping strategies. DV = Dependent variables. The DVs that need to be estimated for each of the *n*_*Fermian*_ subtasks of the similarity-based and the butchering strategy are the same—and the same as for the main task. In contrast, the DVs of the chaining strategy need to be different. *S* and *F* can be conceived of as ‘dependent variables’ too, however, we reserve the abbreviation DV for those estimated in *n*_*Fermian*_ subtasks.

While the discussion above emphasizes differences between strategies by focusing on relative weaknesses of Hegelian dialectical-bootstrapping versus Fermian approaches, we hasten to add that one can see both types of approaches fitting into an overarching framework. Particularly, Fig. 6 illustrates how bootstrapping, a term used by H&H, could serve as an umbrella for both Hegelian and Fermian guesstimation.

Bootstrapping means to re-estimate. As H&H point out, the term ‘dialectical’ in their bootstrapping approach “…refers to the Hegelian process of development, which has three stages: thesis (first estimate [*F*]), antithesis (dialectical estimate [*S*]), and synthesis (aggregation [averaging *F* with *S*])” (^6^, p. 231). Consistent with the Hegelian logic that comes with the “anti”, H&H also referred to the strategy they actually tested in their experiment—namely to ask participants to consider that the first estimate was off the mark—as “consider-the-opposite” (^6^, e.g., p. 234).

Yet, one may argue that dialectical-bootstrapping should not be restricted to the consider-the-opposite approach. As H&H specify: “We do not confine dialectical-bootstrapping to the consider-the-opposite strategy. Rather, we suggest that any elicitation procedure that taps into somewhat nonredundant, yet plausible knowledge is potentially capable of eliciting effective dialectical estimates” (^6^, p. 236; see also^4^, p. 258). Such a procedure could be, for instance, to ask oneself, after having generated an own estimate (*E*_self_), which estimate another person with whom one often disagrees would have given (*E*_other_)^13^, thereby making use of what^16^ referred to as “perspective taking”. To the extent to which *E*_self_ and *E*_other_ tap into conflicting knowledge and hence could be seen as thesis and anti-thesis, respectively, this strategy may qualify as dialectic. However, and in contrast to consider-the-opposite, in which *S* requires *F*, *E*_other_ does not require *E*_self_. It would even be possible to elicit *E*_other_ alone—but it would be impossible to ask for an anti-thesis without having a thesis as a starting point.

One can think of our study as focusing on a way to bootstrap that is *not* dialectical (see Fig. 6 and see Box 2 for further discussion), that does *not* assume that the first estimate *F* was off the mark, and that permits ignoring *F* entirely (just like *E*_other_ permits ignoring *E*_self_ entirely). In fact, *F* is not part of Fermian bootstrapping (§6). Rather, Fermian guesstimation estimate *S* by decomposition and subsequent aggregation, following the logic of Fermian back-of-the-envelope calculation that different independent errors in estimating the DVs to *n*_*Fermian*_ subtasks may cancel each other out.

Analogously to how there may be different strategies for dialectical-bootstrapping, also Fermian bootstrapping comes with different strategies (see Box 3 for a discussion of the term ‘Fermian’). We can conceive of at least three candidate strategies, a *similarity-based strategy*, a *butchering strategy*, and a *chaining strategy*, which differ in how *S* is derived: When making estimates for an object (e.g., ox), Fermian problem-decomposition can entail (1) coming up with guesses of the same target-variable (e.g., body-weight) for similar objects (e.g., horse, bear, deer) and subsequently *averaging* those, or (2) estimating parts (e.g., torso, legs, head) that, together, form the whole, and subsequently *adding* those, or (3) estimating parameters in a chain. A famous example of the latter is estimating the number of piano tuners working in Chicago, which can be done by *multiplying* the estimated number of inhabitants, the estimated number of pianos per 1,000 inhabitants, the estimated to-be-tuned pianos per time interval, the estimate of working hours of tuners, and so on. An example of butchering is to estimate the volume of a planet that comes with an irregular shape—namely by breaking it down into smaller cubes^35^. Our Fermian-condition encouraged only the first mechanism, with the similarity-rule likely prompting exemplar-based reasoning^19^. Future studies could examine which of the three types of decomposition fares best, and also combine them, in line with the possibility that individuals may make both exemplar-based and rule-based judgments (e.g.,^20,21^).

Likewise, future studies could also examine how well strategies fare that blend dialectical and Fermian bootstrapping, making the most of both approaches. As Fig. 6 shows, consider-the-opposite does not specify, as a strategy, how *F* is generated. On the other hand, none of the three Fermian-strategies discussed above explicitly incorporates what common sense knows to be a healthy logic of doubting and reexamining one’s conclusions: Not jumping to (false) conclusions is integral part of dialectical reasoning. It may thus be particularly interesting to instruct people to use any of the three Fermian-strategies to estimate *F*, and then consider-the-opposite to estimate *S*. In addition to making use of potentially different, independent knowledge for generating answers to *n*_*Fermian*_ subtasks in *F*, the independence of estimates of *F* and *S* may, this way, be increased by differences in mechanisms that process the knowledge on which *F* and *S*, respectively, are built. Multi-strategy *blending* approaches have been discussed in the literature as alternatives to toolbox approaches; in contrast to the former, the latter assume “that the outputs of different strategies are blended into a joint, hybrid response (i.e., ‘wisdom of strategies’ in one mind)” (^22^, p. 233). At the same time, a diversity of strategies has been reported to enhance the wisdom-of-crowds effect, albeit in group rather than in individual performance^23,46^.

Moreover, instead of equally weighting *F* and *S*, as implied by computing their average, it may be possible to estimate optimal weights of *S* relative to *F,* perhaps also by making people’s confidence in their *F* and *S*, respectively, part of the integration mechanism (see^36^). Differential weighting of *F* and *S* would permit addressing weaknesses (§2 and §3), both in the ‘pure’ dialectical approach adopted in H&H and in the current study as well as in a strategy that blends Fermian estimation of *F* with dialectical-bootstrapping for *S*.

Finally, future studies could also address limitations of the current experimental design: Fermian estimation requires that a problem can be decomposed into *n*_*Fermian*_ ≥ 2 subtasks. For some animals, it could be that participants do not have sufficient knowledge to generate 2 subtasks. Other animals, in turn, might allow for *n*_*Fermian*_ > 2 rather than the *n*_*Fermian*_ = 2 we enforced. Had we (a) enforced using the Fermian-strategy only when subtasks can be generated, and (b) allowed for *n*_*Fermian*_ ≥ 2 (§11), performance might have been higher. Moreover, the Fermian-strategies’ potential could be assessed further by instructing participants to produce Fermian estimates already for *F*, allowing to test those Fermian *F* against the conditions we realized: (a) averages of naïve *F* and dialectical-bootstrappers’ *S*, and (b) averages of naïve *F* and Fermian *S*.

In a section above we referred to ‘predilected areas of applicability’ for the different strategies. Indeed, in line with ecological approaches to human decision making and rationality (e.g.,^37,38^), we believe that performance depends on the match between the minds’ tools and task-environments—that is, the tool’s *ecological rationality* (e.g.,^39^; see e.g.,^7^ on the ecological rationality of averaging *F* and *S*; see also^4^ for a discussion of ecological rationality of the wisdom-of-crowds across people). For example, Fermian similarity-based strategy can only be applied if sufficiently similar objects can be retrieved. Fermian butchering requires decomposability of objects. Such decomposability is given, for instance, when estimating the number of a university’s employees by adding up the estimates for its various faculties, and it may arguably also be possible for the animals featured as stimuli in the current experiment, but it is hard to see how historical dates, as used by H&H in their original study, could be dissected into small elements, constituting a whole. The Fermian chaining strategy, in turn, requires contextual knowledge beyond the target variable, for instance, that a distance between two cities could be calculated (and hence also estimated) by multiplying the (estimated) average speed of a connecting train by its (estimated) travel time. Dialectical strategies such as consider-the-opposite hinges on remembering first estimates, alongside the “assumptions and considerations [that led to the first estimates but that] could have been wrong” (^6^, p. 234). Individuals with strongly impaired memories—be it due to passing of time (i.e., forgetting by memory decay), interference (i.e., forgetting by interference from taxing contexts), or mental illness may not be able to apply this strategy, although one may speculate that moderate memory loss may, reversely, foster the independence of *F* and *S* as well (see also^5^).

To give a final example of how the interplay between task-environments and minds may affect performance: properties of living beings—such as gestation periods of animals—often come with statistical fluctuations over individuals, and possibly in time and geographical region. Such properties represent population means that vary across time and space, implying that they can only be estimated from samples, which, in turn, requires defining reference classes from which such samples are drawn. In contrast, other stimuli come with unique and precisely defined true values; an example is the years of historical events used as stimuli by H&H. Because Fermian-strategies specify mathematical operations for generating *S* (Fig. 6), using those strategies may afford both calculating estimates of population means and, by systematically varying the input into a strategy, estimating different *S* as upper and lower ranges for a mean’s true value.

It may only be once we understand how the characteristics of dialectical, Fermian, and other “simple mental tool[s]” (^6^, p. 236) interplay with the task-environment and with attributes of the tool-users that we will be able to grasp how well the tools perform. Until then we may be left wondering how much wisdom there *really is* in the wisdom of inner crowds.

Box 1: A mechanism of Hegelian dialectical-bootstrapping: Consider-the-opposite H&H^6^ (p. 234) specified their consider-the-opposite strategy as follows:

“[Step 1]: First, assume that your first estimate is off the mark.

[Step 2]: Second, think about a few reasons why that could be. Which assumptions and considerations could have been wrong?

[Step 3]: Third, what do these new considerations imply? Was the first estimate rather too high or too low?

[Step 4]: Fourth, based on this new perspective, make a second, alternative estimate.”

Box 2: Hegelian dialectical-bootstrapping and Fermian bootstrapping: Siblings, cousins, or strangers? A reviewer of a previous version of this article pointed out that H&H introduced dialectical-bootstrapping as a “general framework” (see^4^, p. 258, for that view on H&H), and that it would be incorrect to equate dialectical-bootstrapping with the consider-the-opposite strategy. Rather, the latter strategy is merely “one plausbile approach to elicit…nonredundant estimates” (^4^, p. 258). We agree that H&H’s more general framework allows for potentially different dialectical-bootstrapping strategies and visualized this possibility by empty boxes in Fig. 6. That reviewer further argued that both H&H’s consider-the-opposite and the Fermian approach introduced in this article “are instances of dialectical techniques”. We disagree. In our view, the Fermian approach introduced in this article does not qualify as ‘dialectical’. Rather, we prefer to reserve the term ‘dialectical’ for strategies that actually follow a dialectical logic of thesis, anti-thesis, and synthesis, which is not part of any of the mechanism of the three Fermian-strategies discussed here (Fig. 6). However, we do agree that Fermian guesstimation can be considered as an instance of bootstrapping, and occasionally we also used the term Fermian bootstrapping. Likewise, perspective-taking and estimating public opinion^16,36^ would, in our view, constitute an example of bootstrapping—in the sense of re-estimating, in general, and coming up with nonredundant estimates, in particular—but it would, strictly speaking, not be a good example of dialectical-bootstrapping as it lacks the logic of thesis/anti-thesis. Would perspective-taking be an example of a Fermian strategy? Well, adapting the perspective of one other person lacks task decomposition and subsequent aggregation, but taking multiple perspectives and then averaging the resulting estimates has these features.

That said, it is possible to imagine yet other strategies, including, for instance, blends between Fermian chaining used to generate *F*, followed by a dialectical mechanism that treats the Fermian *F* as a thesis and then searches for flawed reasoning in the construction of the chain to come up with an antithetical *S*. At the end of the day, whether such a strategy—or any other, for that matter—is then called Fermian, dialectical, or something else, is secondary. To speak with^40^, “…the explanatory burden is carried by the nature of the proposed mechanisms and their interactions, not by what they are called” (p. 121). For instance^41^ features decomposition, including both multiplicative and additive (called segmentation there), notably without any reference to Fermi.

Box 3: Why is the similarity-based strategy examined in this article attributed to Enrico Fermi? Prior to running our experiment, a colleague with a background in physics had coincidentally pointed us to Fermi, who had a knack of making simple back-of-the-envelope calculations, akin to simple strategies in the decision sciences ^37^. Indeed, although Fermian guesstimation seems to have drawn surprisingly little attention in the field of judgment-and-decision-making, Fermi problems are discussed in different fields, including education, physics, and AI^35,42,43^. The best-known example of Fermian guesstimation probably comes with the first test of a nuclear bomb in 1945, in the course of which Fermi estimated the explosive yield “… to be about 10 kt by dropping small pieces of paper and observing their motion in the blast wave” (^44^, p. S326). Another well-known example is more central to the current article and cited above: estimating the number of piano-tuners^17,18^ by what we call the chaining strategy. From the piano-tuner example it becomes clear that a key feature of Fermian guesstimation is built in in all three Fermian-strategies discussed here—namely the trick of decomposition and aggregation, be it through averaging (similarity-based strategy) or adding (butchering strategy) or multiplying (chaining strategy). Moreover, all three strategies operate by generating, after decomposition into *n*_Fermian_ ≥ 2 subtasks, *n*_Fermian_ ≥ 2 estimates of DVs to be aggregated to compute S, as in the well-known piano player example. Finally, all three strategies afford simple back-of-the-envelope calculation.

### Supplementary Information

Supplementary Information.

## Data Availability

The study reported in the article was not preregistered. The data (except for part 3; see Supplementary Information) can be found at https://osf.io/kwdfg/?view_only=c3b7a7ada7804768a9b8dcff71ab0311.

## Code availability

The code for the analysis has been written in R^45^ and can be found at https://osf.io/kwdfg/?view_only=c3b7a7ada7804768a9b8dcff71ab0311.
