# Supplementary material for: Fermian guesstimation can boost the wisdom-of-the-inner-crowd
Source: Sci Rep. 2024 Feb 29;14:5014. doi: 10.1038/s41598-024-53639-3 (PMC10904748; doi:10.1038/s41598-024-53639-3)
Supplement: Supplementary file 1 — Supplementary Information. [file 41598_2024_53639_MOESM1_ESM.docx]

**Supplementary Information**

**Fermian guesstimation can boost the wisdom-of-the-inner-crowd**

Tamara Gomilsek, Ulrich Hoffrage, & Julian N. Marewski

Faculty of Business and Economics

Department of Organizational Behavior

University of Lausanne

Correspondence should be addressed to Tamara Gomilsek ([tamara.gomilsek@gmail.com](mailto:tamara.gomilsek@gmail.com)) or Ulrich Hoffrage ([ulrich.hoffrage@unil.ch](mailto:ulrich.hoffrage@unil.ch)).

Table of Content

[**Section A:** Estimation Questions and True Values 1](#_Toc154172779)

[**Section B:** Experimental Procedure 12](#_Toc154172780)

[Part 1: First estimation 12](#_Toc154172781)

[Part 2: Second estimation 12](#_Toc154172782)

[Part 3: Pair-comparison task 12](#_Toc154172783)

[Part 4: Follow-up questions 13](#_Toc154172784)

[**Section C:** Screenshots 14](#_Toc154172785)

[**Section D:** Trimming 41](#_Toc154172786)

[Percentages of various constellations between first, second, and averaged estimates 42](#_Toc154172787)

[Relative errors 43](#_Toc154172788)

[Distribution of error-reductions 44](#_Toc154172789)

[Average error-reduction 46](#_Toc154172790)

[Commonalities and differences between item-trimming and participant-trimming 47](#_Toc154172791)

[**Section E:** Descriptive Analyses of Follow-up Questions 49](#_Toc154172792)

[**Section F:** Additional Discussion of the Strategies 58](#_Toc154172793)

[**References** 60](#_Toc154172794)

# Section A: Estimation Questions and True Values

For each of our 50 Estimation Questions, we searched online for answers from two different sources. If the answer was given as a range, we took the midpoint for the answer. Next, we took the average of the answers provided in these two sources as the True Value (Table S1).

Table S1

Animal properties

| **Estimation Question** | **Source 1** | **Answer 1** | **Source 2** | **Answer 2** | **True**  **Value** | **Unit** |
| --- | --- | --- | --- | --- | --- | --- |
| “What is the average gestation period of a female Arabian Horse in the wild?” | Valera, M., Blesa, F., Dos Santos, R., & Molina, A. (2006). Genetic study of gestation length in Andalusian and Arabian mares. *Animal Reproduction Science*, *95*(1-2), 75–96. https://doi.org/10.1016/j.anireprosci.2005.09.008 | 340.3 days | Cilek, S. (2009). The survey of reproductive success in Arabian horse breeding from 1976-2007 at Anadolu state farm in Turkey. *Journal of Animal and Veterinary Advances, 8*(2), 389-396. | 334.3 days | 337.3 days = 48.2 weeks | weeks |
| “What is the average gestation period of a female Grey Wolf in the wild?” | *Basic facts about wolfs* (n.d.). Wolf Heaven International*.* Retrieved August 30, 2021, from https://wolfhaven.org/conservation/wolves/basic-facts-about-gray-wolves/ | 63 days | Smith, J. (2002). *Canis lupus (On-line).* Animal Diversity Lab. Retrieved August 30, 2021, from https://animaldiversity.org/accounts/Canis_lupus/ | 60-63 days = 61.5 days | 62.25 = 8.9 weeks | weeks |
| “What is the average gestation period of a female Plain Zebra in the wild?” | *Plains zebra (Equus quagga) fact sheet: Reproduction & development* (2021, March 9). San Diego Zoo Wildlife Alliance Library. Retrieved August 30, 2021, from https://ielc.libguides.com/sdzg/factsheets/plains_zebra/reproduction | 360-396 days = 378 days | *Plains zebra* (n.d.). The Animal Files. Retrieved August 30, 2021, from https://www.theanimalfiles.com/mammals/hoofed_mammals/zebra_plains.html | 370 days | 374 = 53.4 weeks | weeks |
| “What is the average gestation period of a female Red Fox in the wild?” | Fox, D. (2007). *Vulpes vulpes (On-line).* Animal Diversity Web. Retrieved August 30, 2021, from https://animaldiversity.org/accounts/Vulpes_vulpes/ | 51-53 days = 52 days | *The red fox* (n.d.). Department of Natural Resources, Maryland. Retrieved August 30, 2021, from https://dnr.maryland.gov/wildlife/Pages/plants_wildlife/Red_Fox.aspx | 51-53 days = 52 days | 52 = 7.4 weeks | weeks |
| “What is the average gestation period of a female Asian Elephant in the wild?” | Hildebrandt, T., Drews, B., Gaeth, A. P., Goeritz, F., Hermes, R., Schmitt, D., Gray, C., Rich, P., Streich, W. J., Short, R. V., & Renfree, M. B. (2006). Foetal age determination and developments in elephants. *Proceedings of the Royal Society B, 274,* 323-331. <https://doi.org/10.1098/rspb.2006.3738> | 623-729 days = 676 days | *Asian elephant (Elephas maximus) fact sheet: Reproduction & development* (2021, August 27). San Diego Zoo Wildlife Alliance Library. Retrieved August 30, 2021, from https://ielc.libguides.com/sdzg/factsheets/asianelephant/reproduction | 18-22 months = 547.5-669.2 days = 608.35 days | 642.175 = 91.7 weeks | weeks |
| “What is the average gestation period of a female Short-beaked Dolphin in the wild?” | Westgate, A. J., & Read, A. J. (2007). Reproduction in short-beaked common dolphins (Delphinus delphis) from the western North Atlantic. *Marine Biology*, *150*(5), 1011-1024. | 356.6 days | Danil, K., & Chivers, S. J. (2007). Growth and reproduction of female short-beaked common dolphins, Delphinus delphis, in the eastern tropical Pacific. *Canadian Journal of Zoology*, *85*(1), 108-121. <https://doi.org/10.1139/z06-188> | 11.4 months = 346.75 days | 351.675 = 50.2 weeks | weeks |
| “What is the average gestation period of a female Bengal Tiger in the wild?” | *Royal Bengal tiger* (n.d.). Indira Gandhi Zoological Park. Retrieved August 30, 2021, from https://vizagzoo.com/animal-world/carnivores/ | 104-106 days = 105 days | Banglapedia (n.d). Bengal tiger. *National Encyclopedia of Bangladesh.* Retrieved from https://en.banglapedia.org/index.php/Bengal_Tiger | 14-15 weeks = 98 days-105 days = 101.5 days | 103.25 = 14.8 weeks | weeks |
| “What is the average gestation period of a female Angora Goat in the wild?” | Jordan, R. M., (1990). *Angora goats in the Midwest.* Historical Materials from University of Nebraska-Lincoln Extension, 240. Retrieved August 30, 2021, from https://digitalcommons.unl.edu/extensionhist/240/?utm_source=digitalcommons.unl.edu%2Fextensionhist%2F240&utm_medium=PDF&utm_campaign=PDFCoverPages | 148-150 days = 149 days | Knapp, K. (2021). *Angora goat: breed info, characteristics, breeding, and care.* Morning Chores. Retrieved August 30, 2021, from https://morningchores.com/angora-goat/ | 150 days | 149.5 = 21.4 weeks | weeks |
| “What is the average gestation period of a female Chimpanzee in the wild?” | *Chimpanzee* (n.d.). ZSL Whipsnade Zoo. Retrieved August 30, 2021, from https://www.zsl.org/zsl-whipsnade-zoo/chimpanzee | 202-261 days = 231.5 days | Peacock, L. J., & Rogers, C. M., (1959). Gestation period and twinning in chimpanzees. *Science, 129*(3354), 959. https://doi.org/[10.1126/science.129.3354.959.a](https://doi.org/10.1126/science.129.3354.959.a) | 226.8 days | 229.15 = 32.7 weeks | weeks |
| “What is the average gestation period of a female Giant Panda in the wild?” | *Life cycle* (2020). WWF. Retrieved August 30, 2021, from https://wwf.panda.org/discover/knowledge_hub/endangered_species/giant_panda/panda/panda_life_cycle/ | 95-160 days = 127.5 days | Starr, M. (2019, December 16). *Giant panda cubs are born shockingly small – turns out they´re “undercooked”.* Science Alert. Retrieved August 30, 2021, from https://www.sciencealert.com/all-giant-panda-cubs-are-basically-born-premature-and-that-s-why-they-re-so-small | 97-161 days = 129 days | 128.25 = 18.3 weeks | weeks |
| “What is the average lifespan of a female Arabian Horse in the wild?” | *Arabian horse lifespan – how long do they live?* (n.d.). Horse is Love. Retrieved August 30, 2021, from https://horseislove.com/arabian-horse-lifespan/ | 30-35 years = 32.5 years = 390 months | Beach, H. (n.d.). *How long is the average Arabian horse´s life expectancy?.* Pets on Mom. Retrieved August 30, 2021, from https://animals.mom.com/long-average-arabian-horses-life-expectancy-10612.html | 25-30 years = 27.5 years = 330 months | 360 | months |
| “What is the average lifespan of a female Grey Wolf in the wild?” | *Gray wolf facts* (2012, April 13). Nature. Retrieved August 30, 2021, from https://www.pbs.org/wnet/nature/river-of-no-return-gray-wolf-fact-sheet/7659/ | 6-8 years = 7 years = 84 months | Smith, J. (2002). *Canis lupus gray wolf (On-line).* Animal Diversity Lab. Retrieved August 30, 2021, from https://animaldiversity.org/accounts/Canis_lupus/ | 5-6 years = 5.5 years = 66 months | 75 | months |
| “What is the average lifespan of a female Plain Zebra in the wild?” | *Plains zebra* (n.d.). Animal Spot. Retrieved August 30, 2021, from https://www.animalspot.net/plains-zebra.html | 20 years = 240 months | *Plains zebra* (n.d.). Animal Corner. Retrieved August 30, 2021, from https://animalcorner.org/animals/plains-zebra/ | 20-25 years = 22.5 years = 270 months | 255 | months |
| “What is the average lifespan of a female Red Fox in the wild?” | *Red fox* (2015). National Geographic. Retrieved August 30, 2021, from https://www.nationalgeographic.com/animals/mammals/facts/red-fox | 2-4 years = 3 years = 36 months | *Red fox* (n.d.). Chesapeake Bay Program. Retrieved August 30, 2021, from https://www.chesapeakebay.net/discover/field-guide/entry/red_fox | 3 years = 36 months | 36 | months |
| “What is the average lifespan of a female Asian Elephant in the wild?” | *Asian elephant (Elephas maximus) fact sheet: Reproduction & development* (2021, August 27). San Diego Zoo Wildlife Alliance Library. Retrieved August 30, 2021, from https://ielc.libguides.com/sdzg/factsheets/asianelephant/reproduction | 60-70 years = 65 years = 780 months | *Asian Elephant* (2015). National Geographic. Retrieved August 30, 2021, from https://www.nationalgeographic.com/animals/mammals/facts/asian-elephant | 60 years = 720 months | 750 | months |
| “What is the average lifespan of a female Short-beaked Dolphin in the wild?” | *Short-beaked common dolphin* (2021). Animalia. Retrieved August 30, 2021, from https://animalia.bio/short-beaked-common-dolphin | 22-35 years = 28.5 years = 342 months | *Short-beaked common dolphin* (2021). Ocean Conservation Society. Retrieved August 30, 2021, from https://www.oceanconservation.org/dolphin-whale-facts/short-beaked-common-dolphin/ | 35-40 years = 37.5 years = 450 months | 396 | months |
| “What is the average lifespan of a female Bengal Tiger in the wild?” | *Bengal tiger* (2015). National Geographic. Retrieved August 30, 2021, from https://www.nationalgeographic.com/animals/mammals/facts/bengal-tiger | 8-10 years = 9 years = 108 months | *Royal Bengal tiger* (n.d.). Indira Gandhi Zoological Park. Retrieved August 30, 2021, from https://www.vizagzoo.com/animal-world/carnivores/ | 8-10 years = 9 years = 108 months | 108 | months |
| “What is the average lifespan of a female Angora Goat in the wild?” | Cosgrove, N. (2021). *Angora goat info: Facts, pictures, behavior & care guide.* PetKeen. Retrieved August 30, 2021, from https://petkeen.com/angora-goat/ | 10 years = 120 months | Lambert, A. H. (2020, March 2). *Angora goat origin, facts, lifespan, size, milk production.* SheepaDoodle*.* Retrieve August 30, 2021, from http://www.sheepadoodle.info/2020/03/Angora-Goat.html#point2 | 10 years = 120 months | 120 | months |
| “What is the average lifespan of a female Chimpanzee in the wild?” | Hill, K., Boesch, C., Goodall, J., Pusey, A., Williams, J., & Wrangham, R. (2001). Mortality rates among wild chimpanzees. *Journal of Human Evolution, 40*(5), 437–450. https://doi.org/10.1006/jhev.2001.0469 | 15 years = 180 months | J. B. (2013, March 8). *How long do chimpanzees live?.* Chimpanzee Sanctuary Northwest. Retrieved August 30, 2021, from https://chimpsnw.org/2013/03/how-long-do-chimpanzees-live/ | 15 years = 180 months | 180 | months |
| “What is the average lifespan of a female Giant Panda in the wild?” | *Life cycle* (2021). WWF. Retrieved August 30, 2021, from https://wwf.panda.org/discover/knowledge_hub/endangered_species/giant_panda/panda/panda_life_cycle/ | 14-20 years = 17 years = 204 months | *China panda Facts* (2021). Travel China Guide. Retrieved August 30, 2021, from https://www.travelchinaguide.com/tour/panda/facts.htm | 15-20 years = 17.5 years = 210 months | 207 | months |
| “What is the average body weight of a female Arabian Horse in the wild?” | *Horse breeds height & weight chart* (2021). Horsey Hooves. Retrieved August 30, 2021, from https://horseyhooves.com/horse-breeds-height-weight-chart/ | 380-480 kg = 430 kg = 430000 g | Britannica, T. Editors of Encyclopedia (2023, January 17). Arabian horse. *Encyclopedia Britannica.* https://www.britannica.com/animal/Arabian-horse | 360-450 kg = 405 kg = 405000 g | 417500 | grams |
| “What is the average body weight of a female Grey Wolf in the wild?” | *Gray wolf* (n.d.). The National Wildlife Federation. Retrieved August 30, 2021, from https://www.nwf.org/Educational-Resources/Wildlife-Guide/Mammals/Gray-Wolf#:~:text=The%20average%20size%20of%20a,thirds%20of%20the%20United%20States | 27.125-45.4 kg = 36.26 kg = 36260 g | *How much does a female wolf weigh* (n.d.). Justagric. Retrieved August 30, 2021, from https://justagric.com/how-much-does-a-female-wolf-weigh/ | 22.68-38.55 kg = 30.62 kg = 30620 g | 33440 | grams |
| “What is the average body weight of a female Plain Zebra in the wild?” | *Facts & profile plains zebra Equus quagga* (2021). Bilderreich. Retrieved August, 30, 2021, from https://bilderreich.de/1334/fact-sheet-plains-zebra-equus-quagga.html | 175-250 kg = 212.5 kg = 212500 g | *Plains zebra (Equus quagga) fact sheet: Physical characteristics* (2021). San Diego Zoo Wildlife Alliance Library. Retrieved August 30, 2021, from https://ielc.libguides.com/sdzg/factsheets/plains_zebra/characteristics | 273-387 kg = 330 kg = 330000 g | 271250 | grams |
| “What is the average body weight of a female Red Fox in the wild?” | *Red fox size* (n.d.). Wildlife Online. Retrieved August 31, 2021, from https://www.wildlifeonline.me.uk/animals/article/red-fox-size | 5.5 kg = 5500 g | *Species – fox* (2021). The Mammal Society. Retrieved August 31, 2021, from https://www.mammal.org.uk/species-hub/full-species-hub/discover-mammals/species-fox/ | 5-6 kg = 5.5 kg = 5500 g | 5500 | grams |
| “What is the average body weight of a female Asian Elephant in the wild?” | *Asian elephant* (n.d.). Denver ZOO. Retrieved August 31, 2021 from https://denverzoo.org/wp-content/uploads/2018/09/Asian-Elephant.pdf | 3465 kg = 3465000 g | *Asian elephant (Elephas maximus) fact sheet: Physical characteristics* (2021). San Diego Zoo Wildlife Alliance Library. Retrieved August 31, 2021, from https://ielc.libguides.com/sdzg/factsheets/asianelephant/characteristics | 2000-5500 kg = 3750 kg = 3750000 g | 3607500 | grams |
| “What is the average body weight of a female Short-beaked Dolphin in the wild?” | *Short-beaked common dolphin (Delphinus delphis)* (n.d.). Dolphins-world. Retrieved August 31, 2021, from https://www.dolphins-world.com/short-beaked-common-dolphin/#:~:text=Weight%20and%20size.,weight%20is%20100%2D136%20kilograms | 100-136 kg = 118 kg = 118000 g | *Short-beaked common dolphin* (2021). Noaa Fisheries. Retrieved August 31, 2021, from https://www.fisheries.noaa.gov/species/short-beaked-common-dolphin#:~:text=Short%2Dbeaked%20common%20dolphins%20are%20small%2C%20measuring%20less%20than%206,and%20weighing%20about%20170%20pounds. | 77.11 kg = 77110 g | 97555 | grams |
| “What is the average body weight of a female Bengal Tiger in the wild?” | *Royal Bengal tiger* (n.d.). Indira Gandhi Zoological Park. Retrieved August 31, 2021, from https://www.vizagzoo.com/animal-world/carnivores/ | 140 kg = 140000 g | *Tiger weight: Species, life stages & gender differences* (2021). Weight of Stuff. Retrieved August 31, 2021, from https://weightofstuff.com/tiger-weight/ | 65-180 kg = 122.5 kg = 122500 g | 131250 | grams |
| “What is the average body weight of a female Angora Goat in the wild?” | *Angora goat characteristics, feeding, breeding* (2021). Roy´s Farm. Retrieved August 31, 2021, from https://www.roysfarm.com/angora-goat/ | 45.36-49.9 kg = 47.63 kg = 47630 g | *Angora goat facts* (n.d.). ThoughtCo. Retrieved August 31, 2021, from https://www.thoughtco.com/angora-goat-4693619#:~:text=Angora%20goats%20are%20small%20in,and%20weigh%20180%E2%80%93225%20pounds. | 31.75-49.9 kg = 40.825 kg = 40825 g | 44227.5 | grams |
| “What is the average body weight of a female Chimpanzee in the wild?” | *Chimpanzee* (2021). Wisconsin National Primate Research Center. Retrieved August 31, 2021, from https://primate.wisc.edu/primate-info-net/pin-factsheets/pin-factsheet-chimpanzee/#:~:text=Chimpanzees%20exhibit%20very%20little%20morphological,lb)%20(Rowe%201996). | 32-47 kg = 39.5 kg = 39500 g | Uehara, S., & Nishida, T. (1987). Body weights of wild chimpanzees (Pan troglodytes schweinfurthii) of the Mahale mountains national park, Tanzania. *American Journal of Biological Anthropology, 72*(3), 315-321. <https://doi.org/10.1002/ajpa.1330720305> | 35.2 kg = 35200 g | 37350 | grams |
| “What is the average body weight of a female Giant Panda in the wild?” | *How much does a giant panda weigh?* (n.d.). BestofPanda. Retrieved August 31, 2021, from https://bestofpanda.com/how-much-does-a-giant-panda-weigh/ | 70-100 kg = 85 kg = 85000 g | *How much does a panda weigh* (2021). Justagric. Retrieved August 31, 2021, from https://justagric.com/how-much-does-a-panda-weigh/ | 70-100 kg = 85 kg = 85000 g | 85000 | grams |
| “What is the average brain weight of a female Arabian Horse in the wild?” | Cozzi, B., Povinelli, M., Ballarin, C., & Granato, A. (2014). The brain of the horse: weight and cephalization quotients. *Brain, Behavior and Evolution*, *83*(1), 9–16. https://doi.org/10.1159/000356527 | 598.63 g | Northrup, J. (2022). *How big is a horse´s brain?.* Animal hearted. Retrieved July 15, 2022, from <https://www.animalhearted.com/blogs/horses/how-big-is-a-horses-brain#:~:text=The%20Equine%20Behavioral%20Health%20Resource,650th%20of%20its%20body%20weight.> | 680-907 g = 793.5 g | 696.065 | grams |
| “What is the average brain weight of a female Grey Wolf in the wild?” | *Exploring data: Graphs and numerical summaries* (n.d.). The Open University. Retrieved July 15, 2022, from https://www.open.edu/openlearn/science-maths-technology/mathematics-statistics/exploring-data-graphs-and-numerical-summaries/content-section-2.6 | 119.5 g | Tartarelli, G., & Bisconti, M. (2006). Trajectories and constraints in brain evolution in primates and cetaceans. *Human Evolution* *21*(3), 275–287. https://doi.org/10.1007/s11598-006-9027-4 | 19.5 g | 69.5 | grams |
| “What is the average brain weight of a female Plain Zebra in the wild?” | Chaumeton, A. S., Gravett, N., Bhagwandin, A., & Manger, P. R. (2020). Tyrosine hydroxylase containing neurons in the thalamic reticular nucleus of male equids. *Neuroanatomy, 110*, 101873. https://doi.org/10.1016/j.jchemneu.2020.101873 | 538.7 g | Burton, R. F. (2006). A new look at the scaling of size in mammalian eyes. *Journal of Zoology, 269*(2), 225-232. https://doi.org/10.1111/j.1469-7998.2006.00111.x | 570 g | 554.35 | grams |
| “What is the average brain weight of a female Red Fox in the wild?” | Tartarelli, G., & Bisconti, M. (2006). Trajectories and constraints in brain evolution in primates and cetaceans. *Human Evolution* *21*(3), 275–287. | 50.4 g | *Survival of the thickest: big brains make mammal populations less dense* (2020, December 23). University of Reading. Retrieved July 15, 2022, from https://archive.reading.ac.uk/news-events/2020/December/pr852897.html | 47 g | 48.7 | grams |
| “What is the average brain weight of a female Asian Elephant in the wild?” | Tartarelli, G., & Bisconti, M. (2006). Trajectories and constraints in brain evolution in primates and cetaceans. *Human Evolution* *21*(3), 275–287. | 4603 g | *Asian elephant (Elephas maximus) fact sheet: Physical characteristics* (2022). San Diego Zoo Wildlife Alliance Library. Retrieved July 15, 2022, from https://ielc.libguides.com/sdzg/factsheets/asianelephant/characteristics | 5500 g | 5051.5 | grams |
| “What is the average brain weight of a female Short-beaked Dolphin in the wild?” | Marino, L. (2009). Cetacean Brains. In L. R. Squire (Ed.), *Encyclopedia of Neuroscience.* Academic Press | 802 g | Marino, L., Sudheimer, K. D., Pabst, D. A., Mclellan, W. A., Filsoof, D., & Johnson, J. I. (2002). Neuroanatomy of the common dolphin (Delphinus delphis) as revealed by magnetic resonance imaging (MRI). *The Anatomical Record, 268*(4)*,* 411-429. https://doi.org/10.1002/ar.10181 | 981 g | 891.5 | grams |
| “What is the average brain weight of a female Bengal Tiger in the wild?” | McNab, B. K., & Eisenberg, J. F. (1989). Brain size and its relation to the rate of metabolism in mammals. *The American Naturalist*, *133*(2), 157–167. http://www.jstor.org/stable/2462294 | 302 g | Gittleman, J. L. (1986). Carnivore brain size, behavioral ecology, and phylogeny. *Journal of Mammalogy*, *67*(1), 23-36. https://doi.org/10.2307/1380998 | 278 g | 290 | grams |
| “What is the average brain weight of a female Angora Goat in the wild?” | Tartarelli, G., & Bisconti, M. (2006). Trajectories and constraints in brain evolution in primates and cetaceans. *Human Evolution* *21*(3), 275–287. https://doi.org/10.1007/s11598-006-9027-4 | 115 g | Briefer, E. F., Haque, S., Baciadonna, L., & McElligott, A. G. (2014). Goats excel at learning and remembering a highly novel cognitive task. *Frontiers in Zoology*, *11*(1), 20. https://doi.org/10.1186/1742-9994-11-20 | 180 g | 147.5 | grams |
| “What is the average brain weight of a female Chimpanzee in the wild?” | Tartarelli, G., & Bisconti, M. (2006). Trajectories and constraints in brain evolution in primates and cetaceans. *Human Evolution* *21*(3), 275–287. https://doi.org/10.1007/s11598-006-9027-4 | 440 g | Herndon, J. G., Tigges, J., Anderson, D. C., Klumpp, S. A., & McClure, H. M. (1999). Brain weight throughout the life span of the chimpanzee. *The Journal of Comparative Neurology*, *409*(4), 567–572. https://doi.org/10.1002/(SICI)1096-9861(19990712)409:4%3C567::AID-CNE4%3E3.0.CO;2-J | 368.1 g | 404.05 | grams |
| “What is the average brain weight of a female Giant Panda in the wild?” | Nie, Y., Speakman, J. R., Wu, Q., Zhang, C., Hu, Y., Xia, M., ... & Wei, F. (2015). Exceptionally low daily energy expenditure in the bamboo-eating giant panda. *Science*, *349*(6244), 171-174. https://doi.org/10.1126/science.aab2413 | 252 g | Gittleman, J. L. (1986). Carnivore brain size, behavioral ecology, and phylogeny. Journal of Mammalogy, *67*(1), 23–36. <https://doi.org/10.2307/1380998> | 235.10 g | 243.55 | grams |
| “What is the average sleep time of a female Arabian Horse in the wild?” | King, A. J. (2022). *Do horses sleep standing up?.* petMD. Retrieved July 15, 2022, from https://www.petmd.com/horse/do-horses-sleep-standing | 5-7 hours = 6 hours = 360 min | *How much do animals sleep?* (n.d.). University of Washington. Retrieved July 15, 2022, from https://faculty.washington.edu/chudler/chasleep.html | 2.9 hours = 174 min | 267 | min |
| “What is the average sleep time of a female Grey Wolf in the wild?” | *How much do wolves sleep?* (n.d.). Justagric. Retrieved July 15, 2022, from https://justagric.com/how-much-do-wolves-sleep/ | 4-10 hours = 7 hours = 420 min | Stapleton, D. (n.d.). *How & where do wolves sleep?.* Retrieved July 15, 2022, from https://misfitanimals.com/wolves/how-do-wolves-sleep/#:~:text=The%20average%20wolf%20sleeps%20between,than%2014%20hours%20a%20day. | 7-8 hours = 7.5 hours = 450 min | 435 | min |
| “What is the average sleep time of a female Plain Zebra in the wild?” | Zebra. (2022, July 15). In *Wikipedia* https://en.wikipedia.org/wiki/Zebra#:~:text=Mountain%20zebras%20can%20be%20found,protection%20against%20flies%20and%20irritation. | 7 hours = 420 min | *Plains zebra (Equus quagga) fact sheet: Behavior & ecology* (2022). San Diego Zoo Wildlife Alliance Library. Retrieved July 15, 2022, from https://ielc.libguides.com/sdzg/factsheets/plains_zebra/behavior | 7 hours = 420 min | 420 | min |
| “What is the average sleep time of a female Red Fox in the wild?” | *Where do foxes sleep? Sleeping behaviors of foxes* (2022). All Things Foxes. Retrieved July 15, 2022, from https://allthingsfoxes.com/where-do-foxes-sleep/#:~:text=Foxes%20in%20the%20wild%20sleep,sleep%20at%20least%208%20hours. | 10 hours = 600 min | Campbell, S. S., & Tobler, I. (1984). Animal sleep: A review of sleep duration across phylogeny. *Neuroscience & Biobehavioral Reviews*, *8*(3), 269-300. https://doi.org/10.1016/0149-7634(84)90054-X | 9.8hours = 588 min | 594 | min |
| “What is the average sleep time of a female Asian Elephant in the wild?” | *Elephants’ sleeping habits explained* (2022). AZ Animals. Retrieved July 15, 2022, from https://a-z-animals.com/blog/elephants-sleeping-habits/#:~:text=Elephants%20are%20some%20of%20the,to%207%20hours%20each%20day. | 2-3 hours = 2.5 hours = 150 min | Walsh, B. (2017). Asian elephant (Elephas maximus) sleep study – long-term quantitative research at Dublin Zoo. *Journal of Zoo and Aquarium Research*, *5*(2), 82–85. https://doi.org/10.19227/jzar.v5i2.174 | 3.33 hours = 199.8 min | 174.9 | min |
| “What is the average sleep time of a female Short-beaked Dolphin in the wild?” | Hecker, B. (1998, February 2). *How do whales and dolphins sleep without drowning?.* Scientific American. Retrieved July 15, 2022, from https://www.scientificamerican.com/article/how-do-whales-and-dolphin/ | 8.016 hours = 480.96 min | Kurtz, K. (2016, March 11). *How do dolphins sleep?.* Retrieved July, 2022, from http://www.kevkurtz.com/his-blog/2016/11/2/how-do-dolphins-sleep | 8 hours = 480 min | 480.48 | min |
| “What is the average sleep time of a female Bengal Tiger in the wild?” | *Things you need to know about Bengal tigers actually* (2020, July 7). All About Animals. Retrieved July 15, 2022, from https://baynamsarf.weebly.com/blog/things-you-need-to-know-about-bengal-tigers-actually | 15.8 hours = 948 min | *Tigers sleep for how many hours? \| Tiger sleeping habits* (n.d.). Tiger Safari in India. Retrieved July 15, 2022, from https://www.pugdundeesafaris.com/blog/tiger-sleeping-habits/#:~:text=Are%20Tigers%20fond%20of%20sleeping,their%20snoozes%20at%20cooler%20places. | 18-20 hours = 19 hours = 1140 min | 1044 | min |
| “What is the average sleep time of a female Angora Goat in the wild?” | *Do goats sleep?* (2021, April 28). Goat Owner. Retrieved July 15, 2022, from https://goatowner.com/do-goats-sleep/ | 5 hours = 300 min | *How much do animals sleep?* (n.d.). University of Washington. Retrieved July 15, 2022, from https://faculty.washington.edu/chudler/chasleep.html | 5.3 hours = 318 min | 309 | min |
| “What is the average sleep time of a female Chimpanzee in the wild?” | *How much do animals sleep?* (n.d.). University of Washington. Retrieved July 15, 2022, from https://faculty.washington.edu/chudler/chasleep.html | 9.7 hours = 582 min | Viegas, J. (2018, March 14). *How human sleep differs from other primates, and what it means for our health.* Animals. Retrieved July 15, 2022, from https://www.seeker.com/animals/how-human-sleep-differs-from-other-primates-and-what-it-means-for-our-health | 9.67-11.5 hours = 10.585 hours = 635.1 min | 608.55 | min |
| “What is the average sleep time of a female Giant Panda in the wild?” | *The pandas sleeping habits (7 things you should know!)* (n.d.). BestOfPanda. Retrieved July 15, 2022, from https://bestofpanda.com/how-many-hours-does-a-panda-sleep/ | 8-12 hours = 10 hours = 600 min | Bhandari, S. (2021, October 5). *How long do pandas sleep (and why)?*. Retrieved July 15, 2022, from https://exactlyhowlong.com/how-long-do-pandas-sleep-and-why/ | 8-12 hours = 10 hours = 600 min | 600 | min |

*Note.* In contrast to H&H who had a precisely defined criterion (year in which historical events happened), our criteria were population means. These can only be estimated based on samples, which, in turn, requires defining reference classes from which such samples are drawn. We tried our best to find such estimates for female animals living in the wild, but we succeeded to find sources with such estimates in approximately only 5% of the cases, and in a small number of cases – maybe 10% – we inferred that the given estimate is more likely to refer to this combination (female and in the wild) than to any other combination. For the rest, either sex (male/female) and/or habitat (wild/caption) was not specified. But even for sources in which estimates for female animals in the wild were given, there was still some arbitrariness, be it because of the choice of the reference class in this source, or because of the sample that has been drawn from this reference class.

# Section B: Experimental Procedure

Participants were welcomed (Figure S1), had to read our consent form and accept the terms (Figures S2-S3), and had to pass a captcha text (identify the pictures with cars in it, no screenshots provided below). Subsequently, the experiment unfolded in four parts. Whereas data from Parts 1, 2, and 4 are reported in the present manuscript, we plan to use the data from part 3 to model participants’ choices (i.e., comparisons between pairs of animals) and to report these analyses elsewhere.

## Part 1: First estimation

In the first part, participants received instructions (Figure S4) for their first estimates. Next, they were provided with 50 Estimation Questions (see Figure S5 for the screenshot and Table S1 for all Estimation Questions). Each of these was, immediately, followed by a Range Question: **“**Specify the lower and the upper limit of your previous estimate such that there is a 90 % chance that the true value falls within this range. Across all 100 ranges, you should have 90 hits (range contains the true value) and 10 misses (range does not contain the true value).” (again, see Figure S5). After these 50 Estimation and Range Questions, participants were led to the second part (Figure S6).

## Part 2: Second estimation

In the second part, participants received one of the three strategy instructions for their second estimates (Figures S7-S9). Next, they were randomly assigned to one of six conditions. In each condition, we presented them again with the exact 50 Estimation Questions (and their Range Questions), albeit in a different random order (see Figures S10-S15). Depending on the strategy condition, participants received different instructions how to provide the second estimate (for details, see main text, section Method/Strategies). Finally, participants were asked for how many items they thought the true values would fall within their ranges (Figure S16). Afterwards, participants were led to the third part (Figure S17).

## Part 3: Pair-comparison task

In the third part, participants received instructions for solving the pair-comparison task (Figure S18). Next, they were presented with all possible pairs (n=45) that could be formed between the ten animals (*Arabian-Horse*, *Grey-Wolf*, *Plain-Zebra*, *Red-Fox*, *Asian-Elephant*, *Short-beaked-Dolphin*, *Bengal-Tiger*, *Angora-Goat*, *Chimpanzee*, *Giant-Panda*). Position of animals within a given pair and order of pairs was determined randomly, and independently for every participant. Participants’ task was to compare a given pair of animals with respect to one of the five properties (*body-weight*, *brain-weight*, *lifespan*, *gestation-period*, *sleep-time*). Each participant was randomly assigned (between-subjects design) to one of the following five Inference Questions (1. “**Which animal has, on average, a longer gestation period?” 2. “Which animal has, on average, a longer sleep time?” 3. “Which animal has, on average, a longer lifespan?” 4. “Which animal weighs more on average?” 5. “Which animal has, on average, a heavier brain weight?”).** A given pair-comparison was immediately followed by a Confidence Question (Figure S19): “Specify how certain you are that your answer is correct by clicking on one of the following confidence categories. To explain the extremes: if your decision was random, you should pick 50 % (and you expect 50 % of all decisions, for which you picked this confidence category, to be correct). If you are absolutely sure that your decision was correct, you should pick 100 % (and you expect 100 % of all decisions, for which you picked this confidence category, to be correct).” Afterward, participants were led to the fourth part (Figure S20).

## Part 4: Follow-up questions

In the fourth part, participants had to first solve the Order Task (Figure S21): “Please rank the following 4 characteristics of animals: average brain weight, average sleep time, average lifespan, and average gestation period by how well you think they can predict average body weight, placing the characteristic that has the greatest predictive power at the top of the list. Note that the 4 characteristics appear in random order.” Next, they tackled the Comparison Task (Figure S22): **“**For the final part of the study, we ask you to specify how body weight is related to other characteristics of animals (e.g., gestation period, sleep time, brain weight, lifespan)? Please use the + sign if you think the relation is positive and the – sign if you think the relationship is negative.” The Order Task question and the Comparison task question referred to the same animal property asked in the pair-comparison task (again, see Figure S19).

Subsequently, participants had to answer the following eight questions (Figures S23-S26):

**1**. “Did you study biology at university, or are you currently a biology student? If yes, please specify which branch of biology?”
**2.** “Do you work with animals? If yes, please specify your job title?”
**3.** “How good is your knowledge of animal species in general? Provide your answer on a scale from 1 to 7, where 1 means you have no knowledge and 7 means you are very knowledgeable.”
**4**. “Can you describe one (or two) of the decision strategies you came up with for the estimations in the first block of the Estimation Task (i.e., when you were asked for the first time to make estimations about each of the animals)? Please be as specific as you can and provide as many details as possible. In your view, when (e.g., under what conditions, in what situations) would your decision strategy work best, and when would it fail?”

**5. “Can we trust your data for scientific research?**

**In case you did not pay attention to the content of the questions or did not follow the instructions properly, or in case there is any other reason why we should exclude your data from our analyses, then please answer "NO". *Please answer honestly; your payment will not be affected by your answer.”**

**6.** “Did you seek any outside help (e.g., Internet, encyclopedia, another individual, etc.) to inform your answers?

If yes, please specify how many Estimation Questions you sought help with. *Please answer honestly; your payment will not be affected by your answer.” **7. “Did you use the fullscreen view during the study?**

**Please answer honestly; your payment will not be affected by your answer.”
8. “Do you have any comments or questions about the study?**

**For instance, (i) were the instructions clear or unclear (if so, which ones and why); (ii) did the questions make sense or not (if so, which ones and why); (iii) did you enjoy the study or not; (iv) were there any mistakes you noticed (which ones)? Anything else?”**

At the end participants were thanked and informed how much money they have earned with this study (Figure S27).

In each part of the experiment, the Qualtrics software (Version [December, 2021] of Qualtrics, Copyright © [2021] Qualtrics) automatically measured and stored timestamps: of the first and the last mouse clicks on a given page, and how long it took participants to provide their answers. The software also stored the number of mouse clicks on a given page. Yet, it could not capture and store any timestamp information from keystrokes. For Parts 1 and 2, we also captured the serial position at which a (randomly placed) item was presented. None of these timestamp and mouse click data were analyzed. We started to run an exploratory analysis on the serial position data but did not pursue it further because we felt it was not relevant to any of our six goals.

# Section C: Screenshots

**Figure S1:** Welcome text

**Figure S2:** Consent form (upper part of the screen)

**Figure S3:** Consent form (lower part of the screen)

**Figure S4:** First estimation task (Part 1)

**Figure S5:** First estimation task, first item

**Figure S6:** End of Part 1

**Figure S7:** Second estimation task (Part 2): Instructions for Dialectical conditions (both for Aided and Unaided)

**Figure S8:** Second estimation task (Part 2): Instructions for Fermi conditions (both for Aided and Unaided)

**Figure S9:** Second estimation task (Part 2): Instructions for Control conditions (both for Aided and Unaided)

**Figure S10:** Second estimation task: Aided-Dialectical-condition, first item

**Figure S11:** Second estimation task: Unaided-Dialectical-condition, first item

**Figure S12.a:** Second estimation task: Aided-Fermi-condition, first item

**Figure S12.b:** Second estimation task: Aided-Fermi-condition, first item (reason for exclusion)

**Figure S13:** Second estimation task: Unaided-Fermi-condition, first item

**Figure S14:** Second estimation task: Aided-Control-condition, first item

**Figure S15:** Second estimation task: Unaided-Control-condition, first item

**Figure S16:** Relative frequency question

**Figure S17:** End of Part 2

**Figure S18:** Pair-comparison task, instructions

**Figure S19:** Pair-comparison task, first item

**Figure S20:** End of Part 3

**Figure S21:** Order task

**Figure S22:** Comparison task

**Figure S23:** Feedback questions 1-4 (upper part of the screen)

**Figure S24:** Feedback questions 1-4 (lower part of the screen)

**Figure S25:** Feedback question 5

**Figure S26:** Feedback questions 6-8

**Figure S27:** Study end

Figure S1

Welcome text


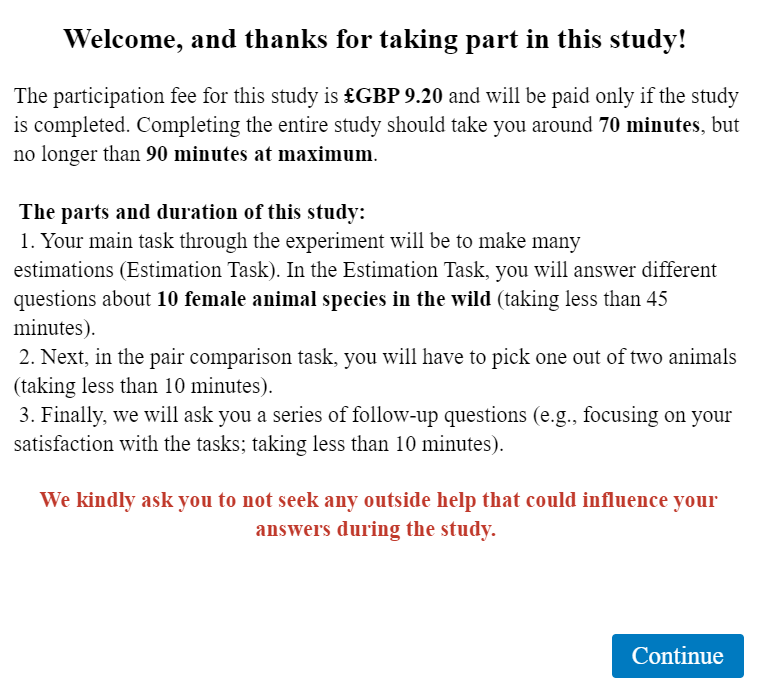


Figure S2

Consent form (upper part of the screen)
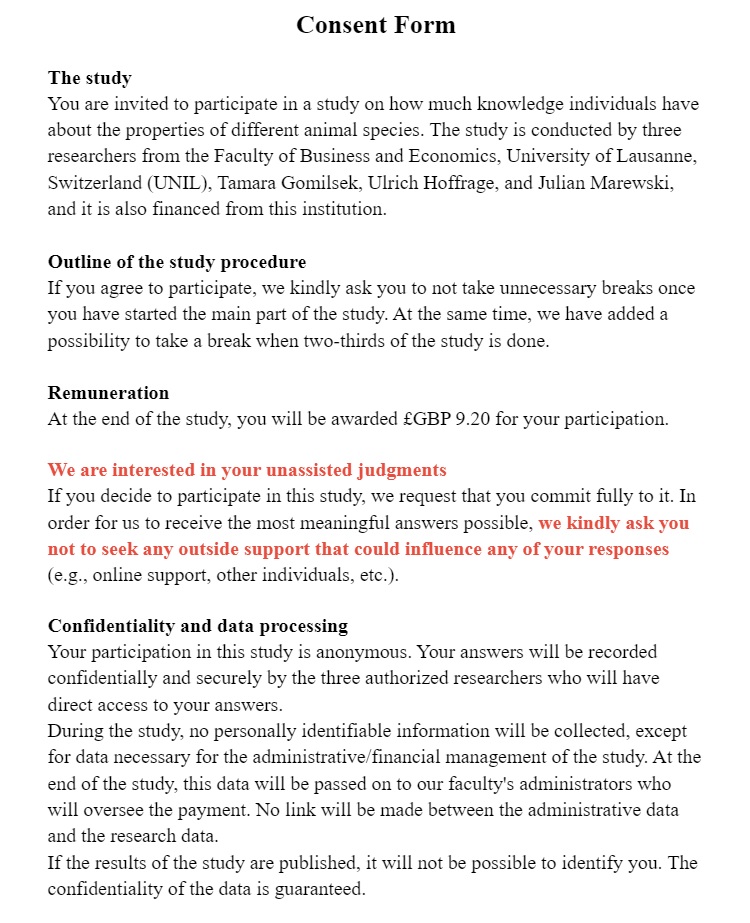


Figure S3

Consent form (lower part of the screen)


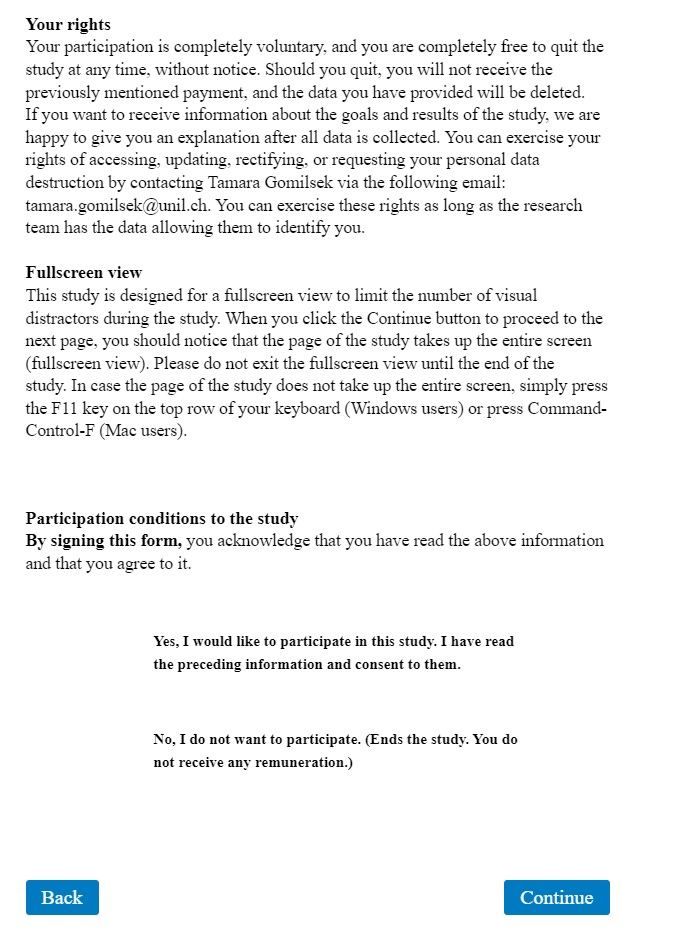


Figure S4

First estimation task (Part 1)
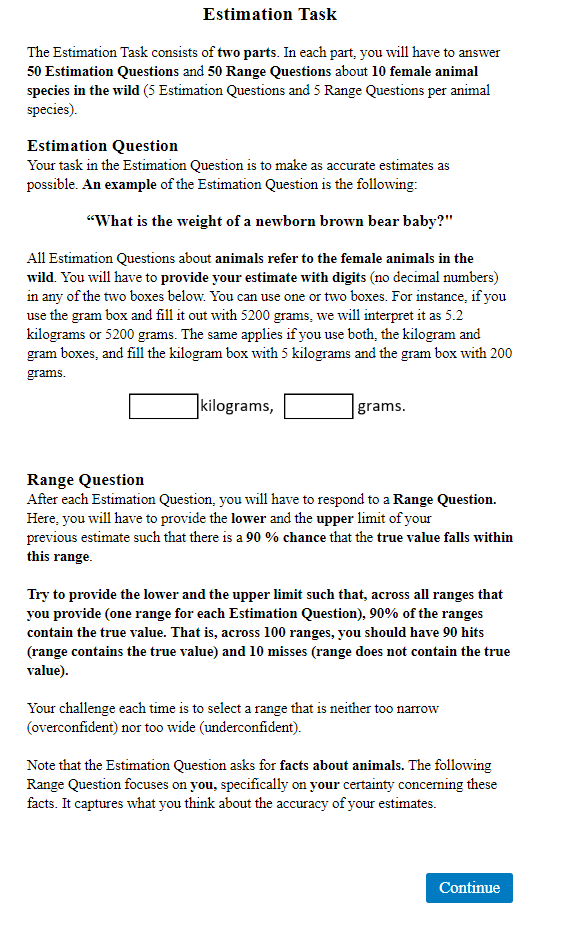


Figure S5

First estimation task, first item


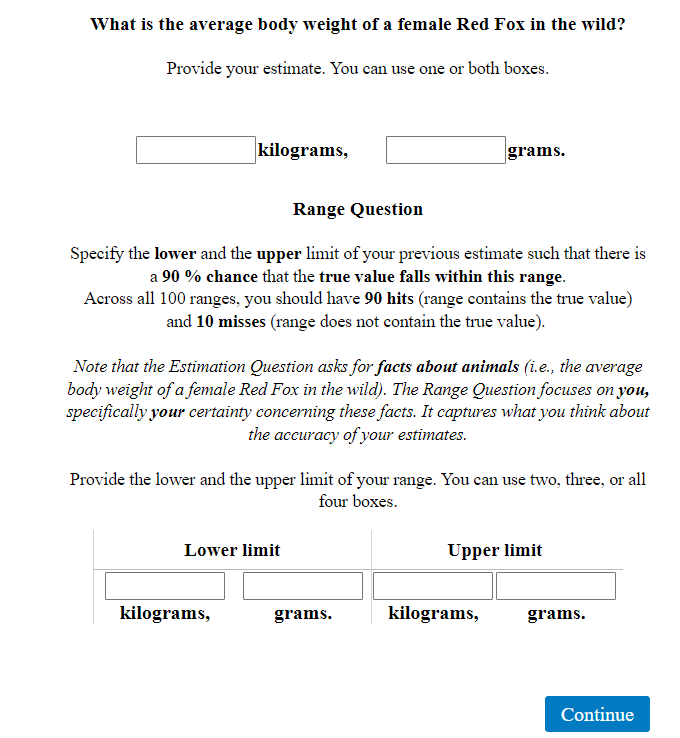


Figure S6

End of Part 1
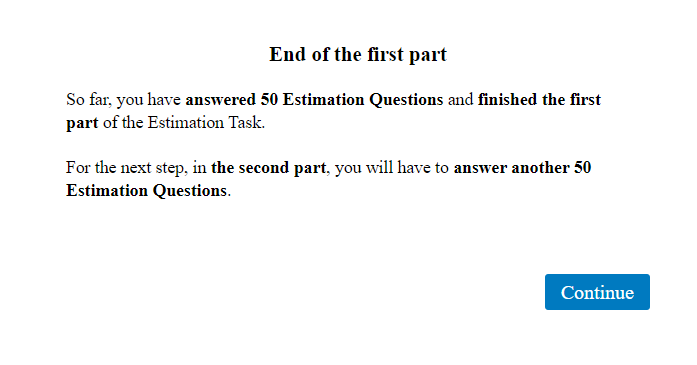


Figure S7

Second estimation task (Part 2): Instructions for Dialectical conditions (both for Aided and Unaided)
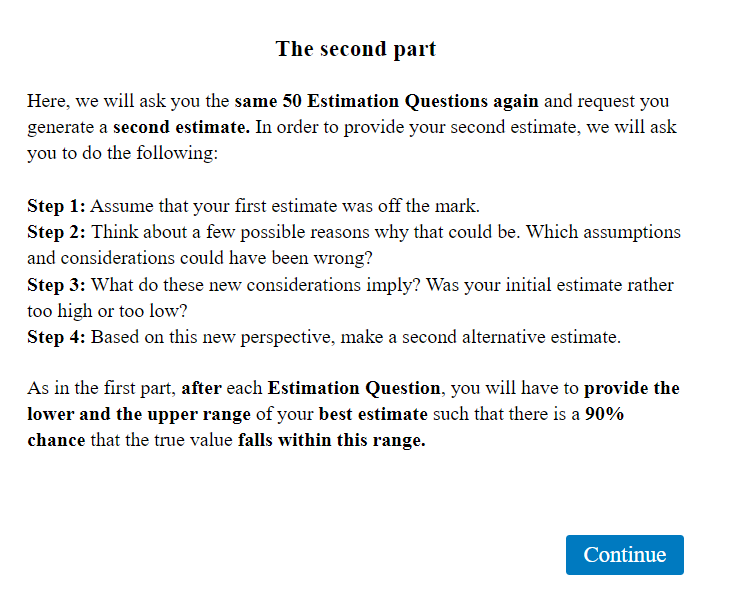


Figure S8

*Second estimation task (Part 2): Instructions for Fermi conditions (both for Aided and Unaided)*


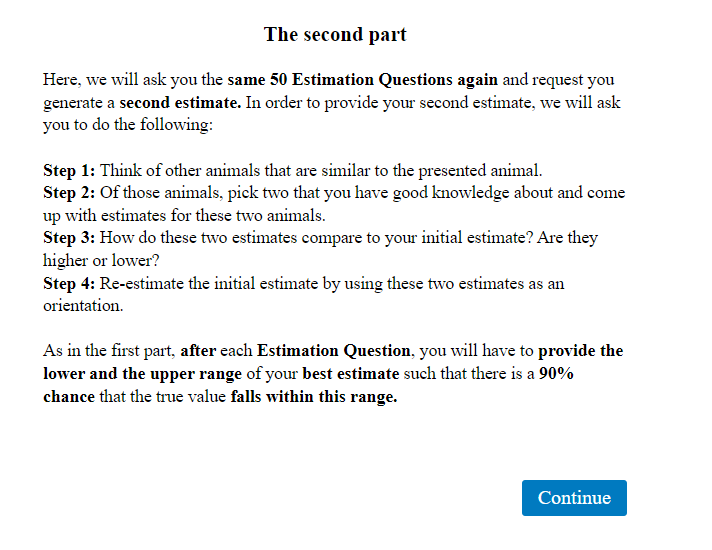


Figure S9

*Second estimation task (Part 2): Instructions for Control conditions (both for Aided and Unaided)*


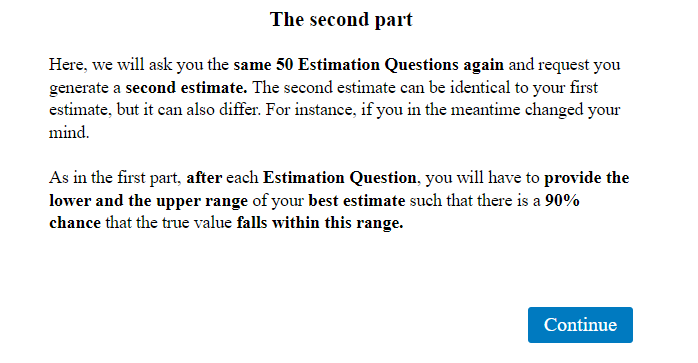


Figure S10

*Second estimation task: Aided-Dialectical-condition, first item*
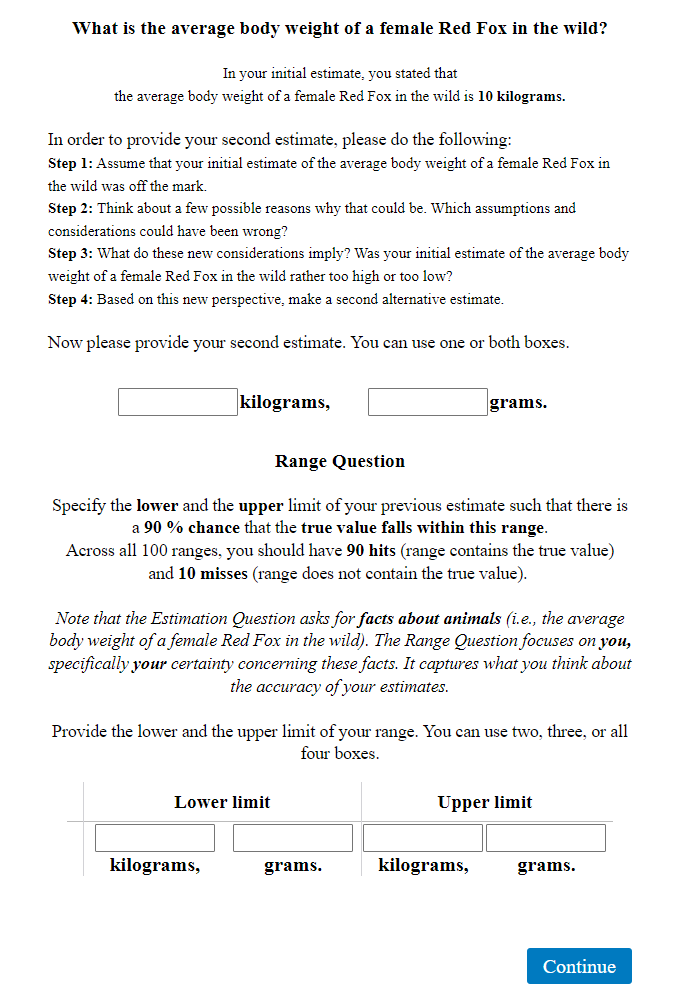


Figure S11

Second estimation task: Unaided-Dialectical-condition, first item
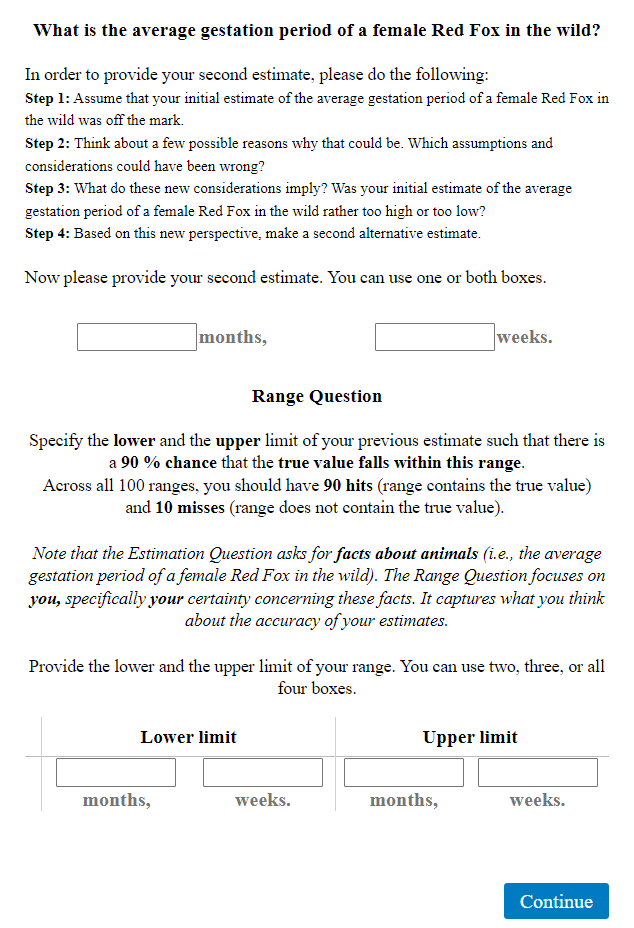


Figure S12.a

*Second estimation task: Aided-Fermi-condition, first item*


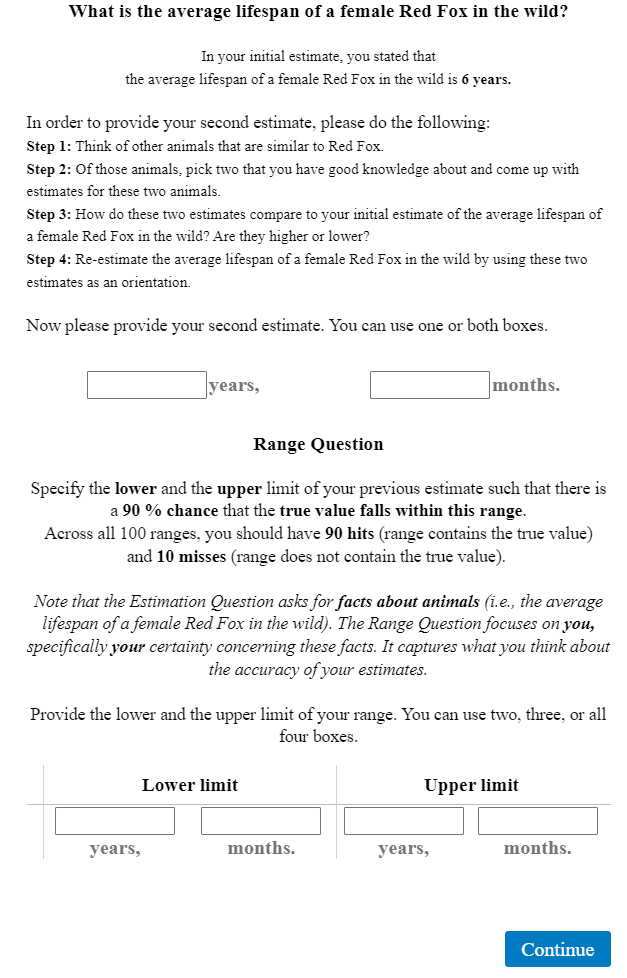


Figure S12.b

*Second estimation task: Aided-Fermi-condition, first item (reason for exclusion)*

As explained in the article (Method, Data-cleaning), there was a software error in the Aided-Fermi-condition that led to the exclusion of all body-weight questions in this condition. The red arrows next to the screenshot below mark the places where the software should have displayed kilograms and grams (instead of tonnes and kilograms, as it actually, and erroneously, did).


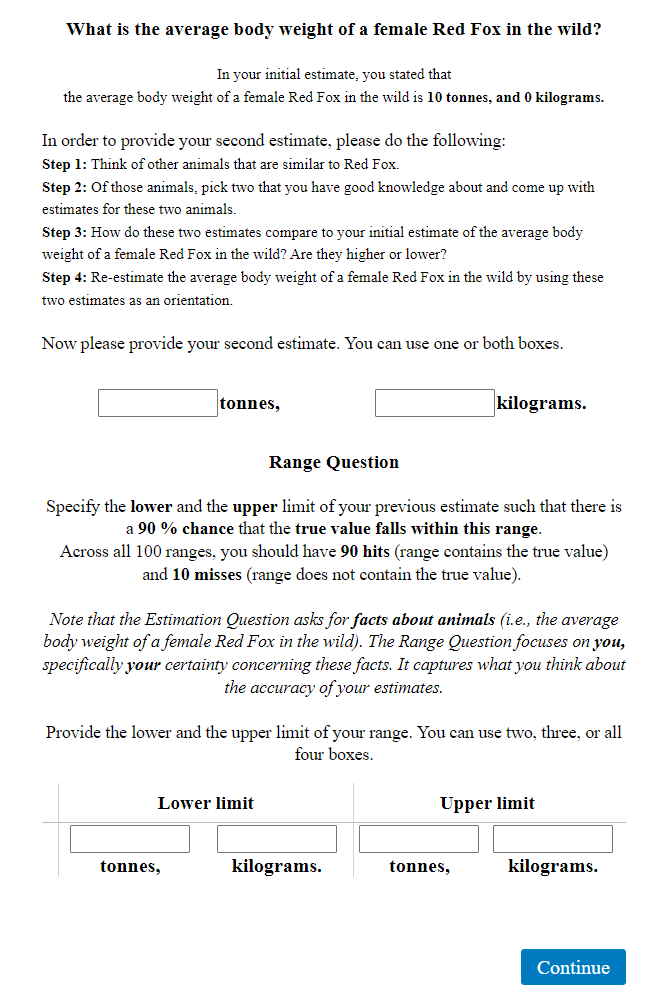


Figure S13

*Second estimation task: Unaided-Fermi-condition, first item*


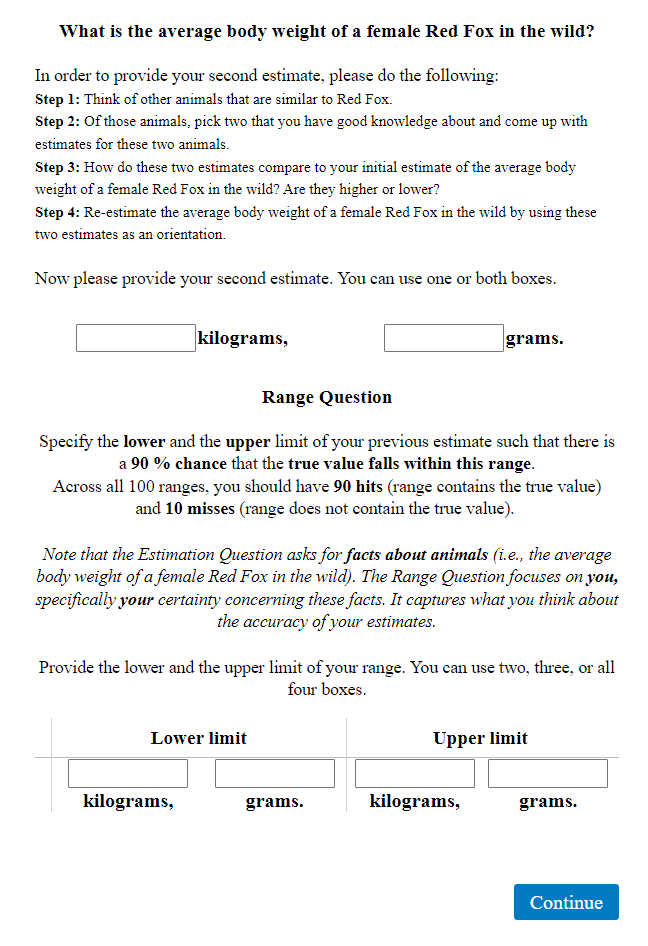


Figure S14

*Second estimation task: Aided-Control-condition, first item*
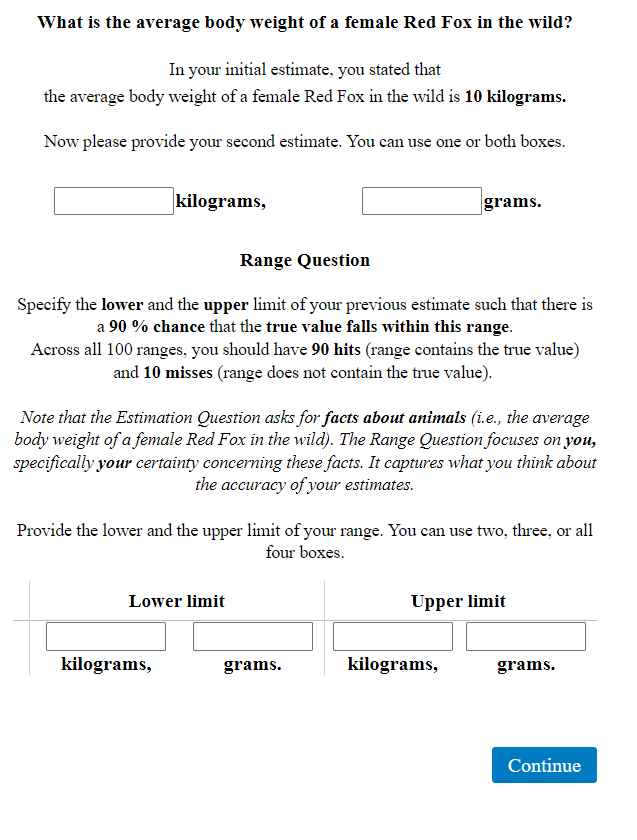


Figure S15

*Second estimation task: Unaided-Control-condition, first item*


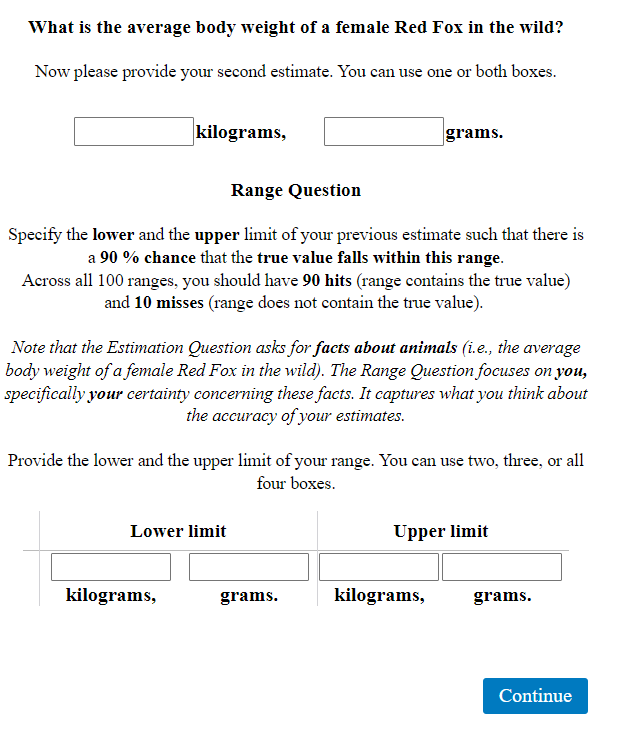


Figure S16

Relative frequency question


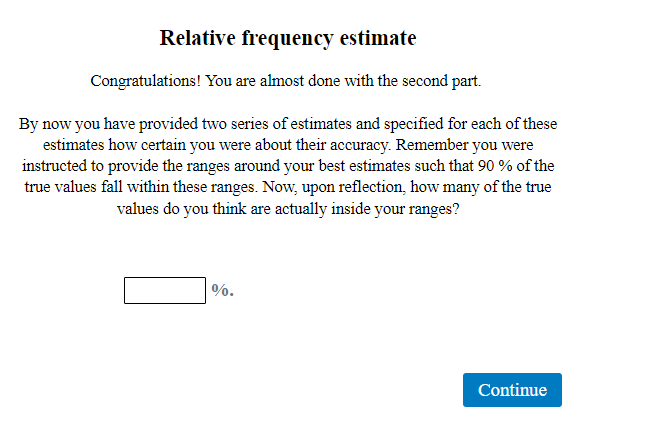


Figure S17

End of Part 2
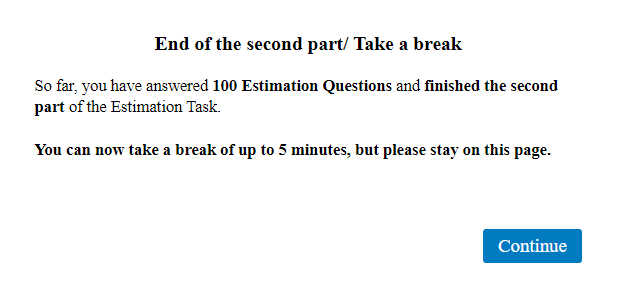


Figure S18

Pair-comparison task, instructions


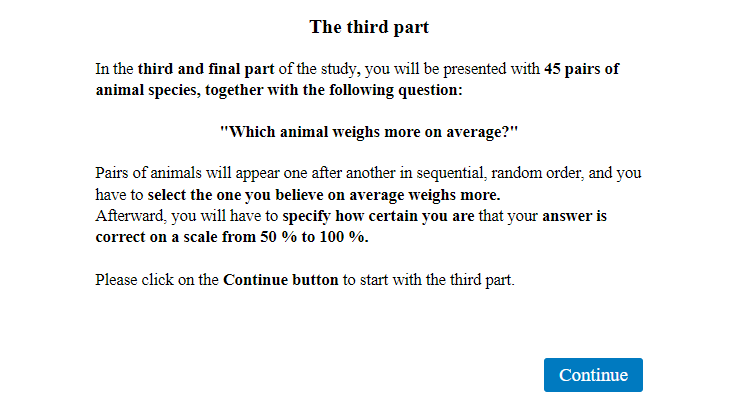


Figure S19

Pair-comparison task, first item


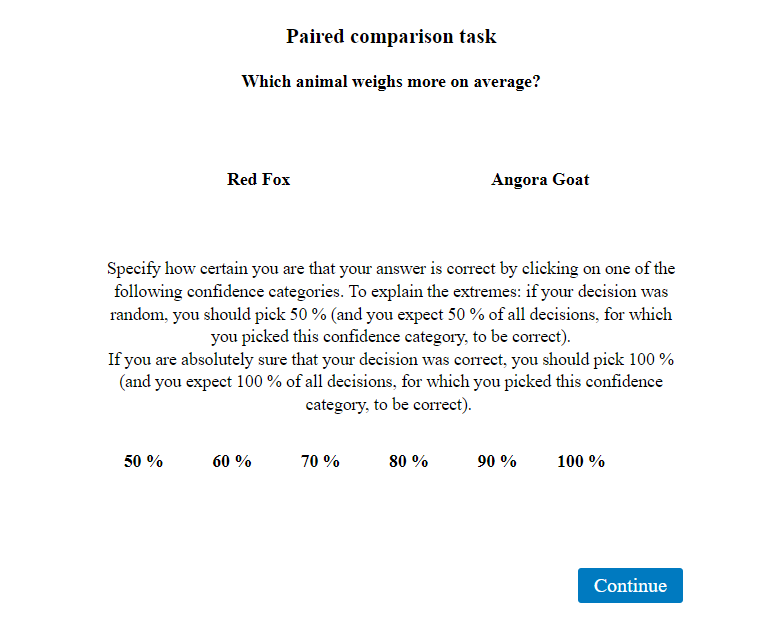


**Figure S20**

End of Part 3


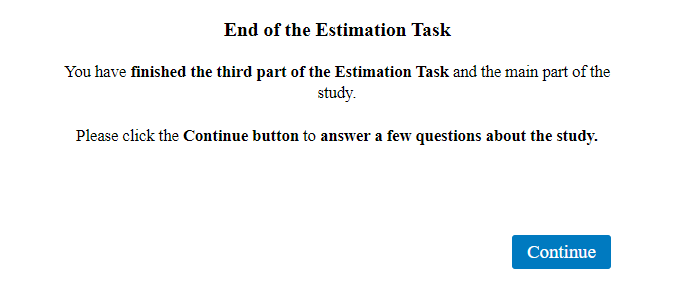


Figure S21

Order task


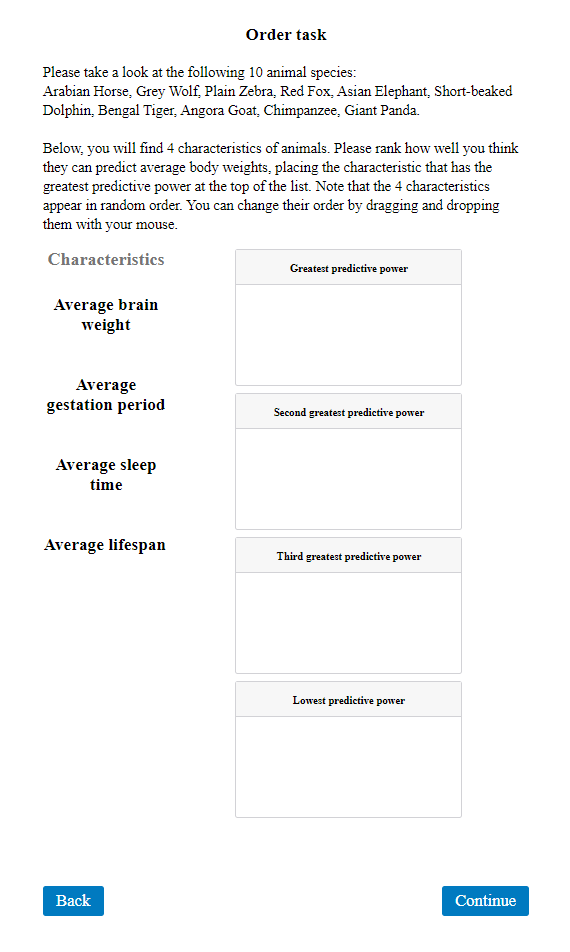


**Figure S22**

Comparison task

**
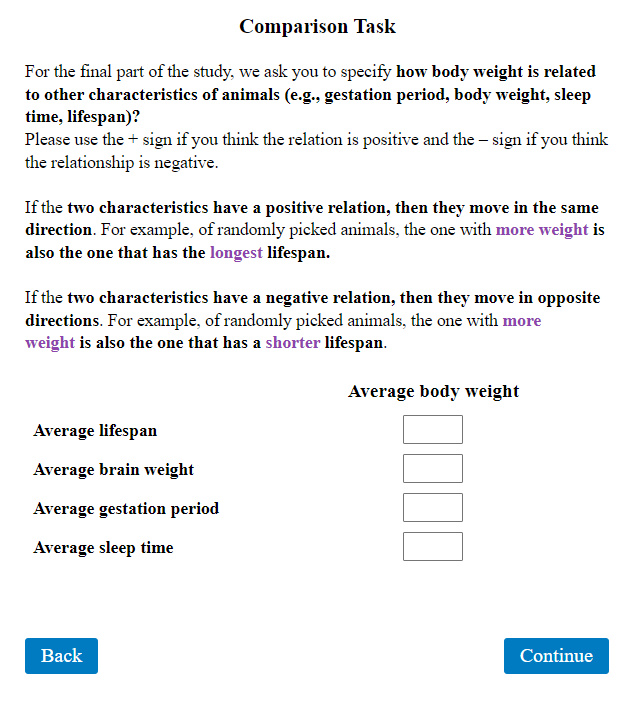
**

Figure S23

Feedback questions 1-4 (upper part of the screen)


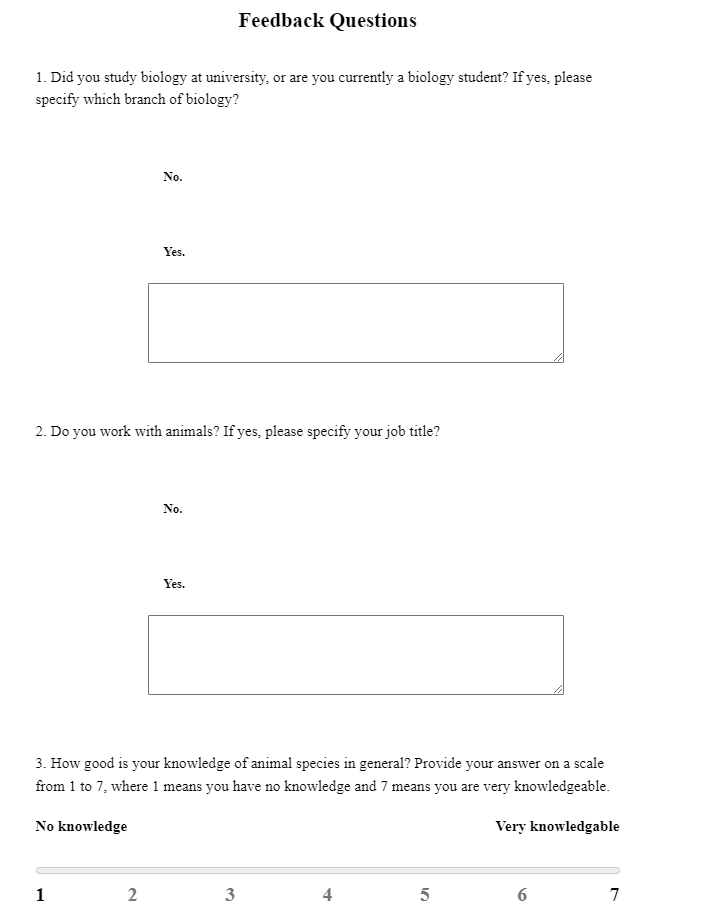


Figure S24

Feedback questions 1-4 (lower part of the screen)


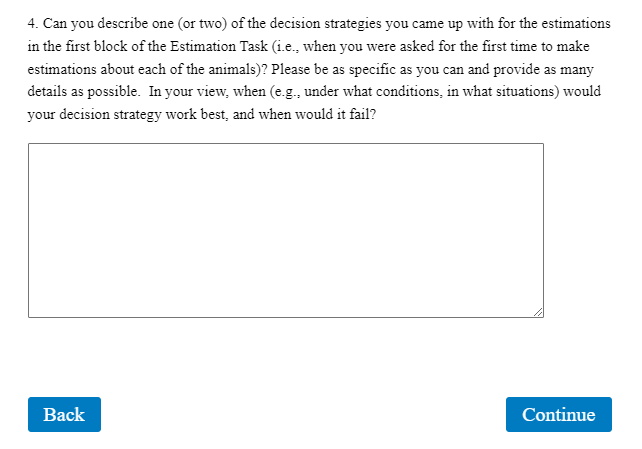


Figure S25

Feedback question 5


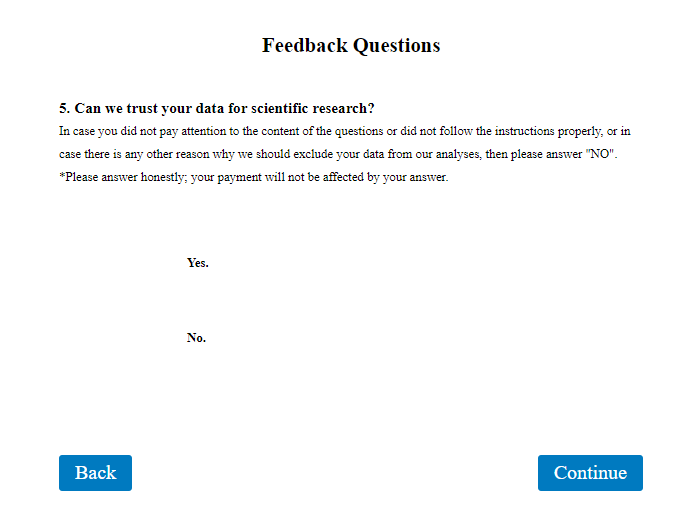


Figure S26

Feedback questions 6-8


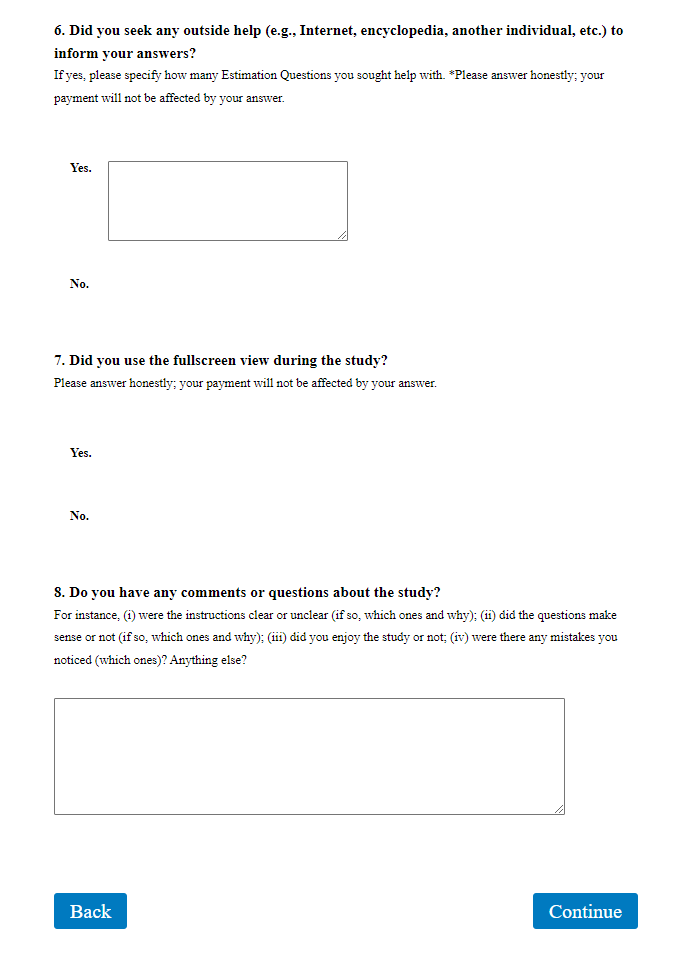


Figure S27

Study end


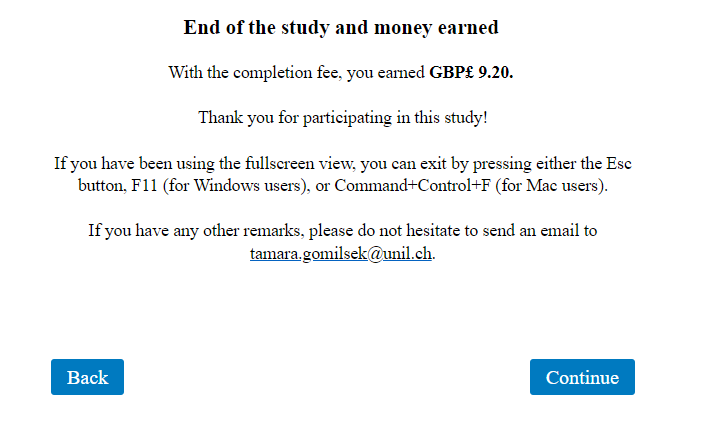


# Section D: Trimming

In the main manuscript, we report 20% trimmed averages, following H&H (see Figs. 1B, 2B, 4B; alongside computing untrimmed averages, Figs. 1A, 2A, 4A). Besides such trimming on the participant-level, we also used data trimming on an item-level, which we realized as follows. First, for each of the 40 items, we ordered all estimates—pooled across *F* and *S*—of all participants of a given condition from lowest to highest. Next, we excluded 20% of the lowest and 20% of the highest estimates. In some cases, the cutoff estimate was provided by more than one participant—in these cases (overall 15.3%), we removed all of them. To ensure that comparisons between first, second, and averaged estimates did not suffer from missing values, we also removed those first (second) estimates for which the corresponding second (first) estimate, made by the same participant, fell into one of the two 20% tails.

In total, the 20% trimming procedure allowed us to keep 41.8% of the estimates (9,526 out of 22,800; 41.2%, 44.9%, 44.5%, 38.0%, 37.4%, and 43.1%, for Aided Dialectical (*AD*), Aided Fermi (*AF*), Aided Control (*AC*), Unaided Dialectical (*UD*), Unaided Fermi (*UF*), and Unaided Control (*UC*), respectively). The median number of usable estimates per participant was 34 out of 80 estimates (*range =* 4 – 68).

In addition to excluding 20% of both tails, we also used 10% and 5%, which led to the exclusion of 58.2%, 32.2%, and 16.5% of the estimates, respectively. In addition, we used 1, 2, and 3 standard deviations (SD) as cutoff criteria, which led to the exclusion of 32.4%, 7.8%, and 4.0% of the estimates. (In the figures below, these exclusion criteria are ordered roughly such that the one that excludes the least data points, 3SD, is displayed next to “All data”, and the one that excludes the most, 20% trim, is displayed at the opposite end.)

As a comparison of the panels in Figs. S28-S31 shows the results and conclusions are quite robust across trimming procedures on the item level (with some limitations, mostly for 20% trim).

## Percentages of various constellations between first, second, and averaged estimates


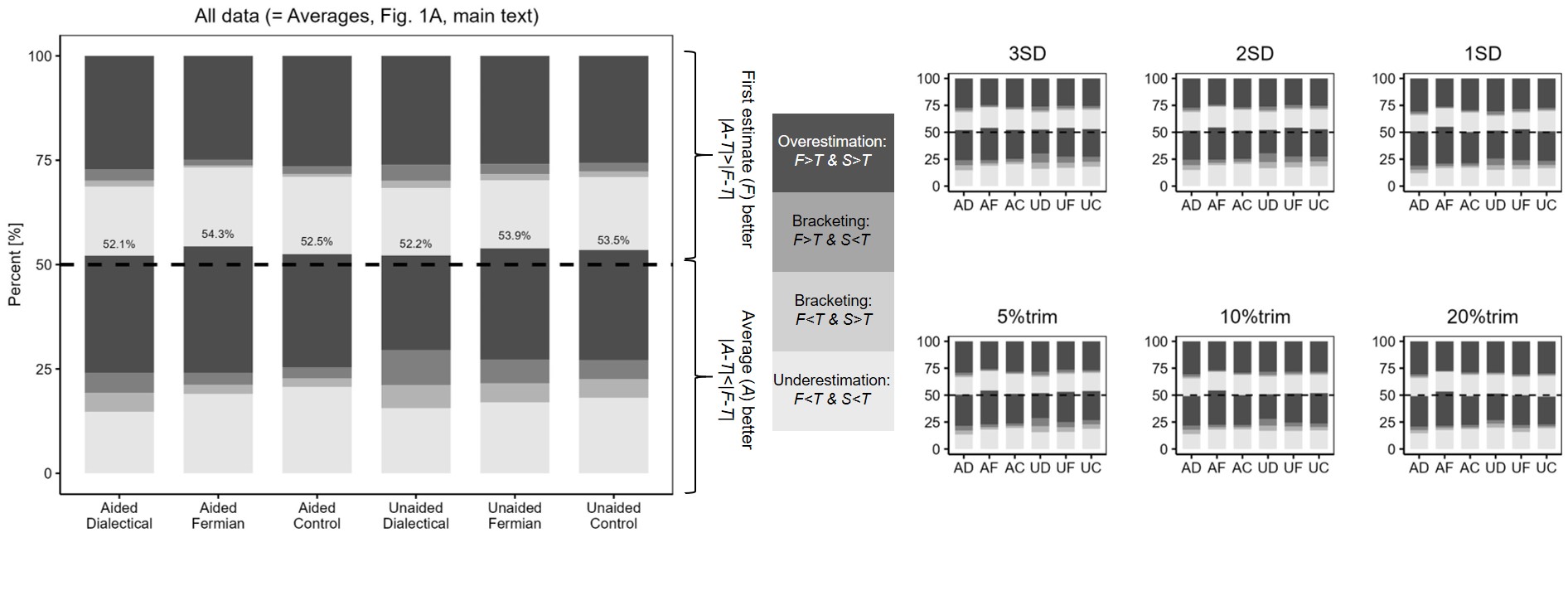


**Fig. S28.** Constellations of First (*F*) and Second (*S*) estimates, and their Average (*A*), with respect to the True value (*T*). Bright bars: *F<T* & *S<T* (underestimation), bright grey: *F<T* & *S>T* (bracketing), dark grey: *F>T* & *S<T* (bracketing), and dark: *F>T* & *S>T* (overestimation). For the four bars at the bottom, below the dashed line, |*A-T*|<|*F-T*|, and for the four bars at the top: |*A-T*|>|*F-T*|. The black number in each condition is the sum of the four bars at the bottom. Fig. S28 (left) displays averages of the proportions across all data and is identical to Fig. 1A in the main text. The six panels to the right display averages of the participant-specific proportions that were computed after excluding items based on various cutoff criteria, namely 3SD, 2SD, 1SD, 5%trim, 10%trim, and 20%trim.

## Relative errors


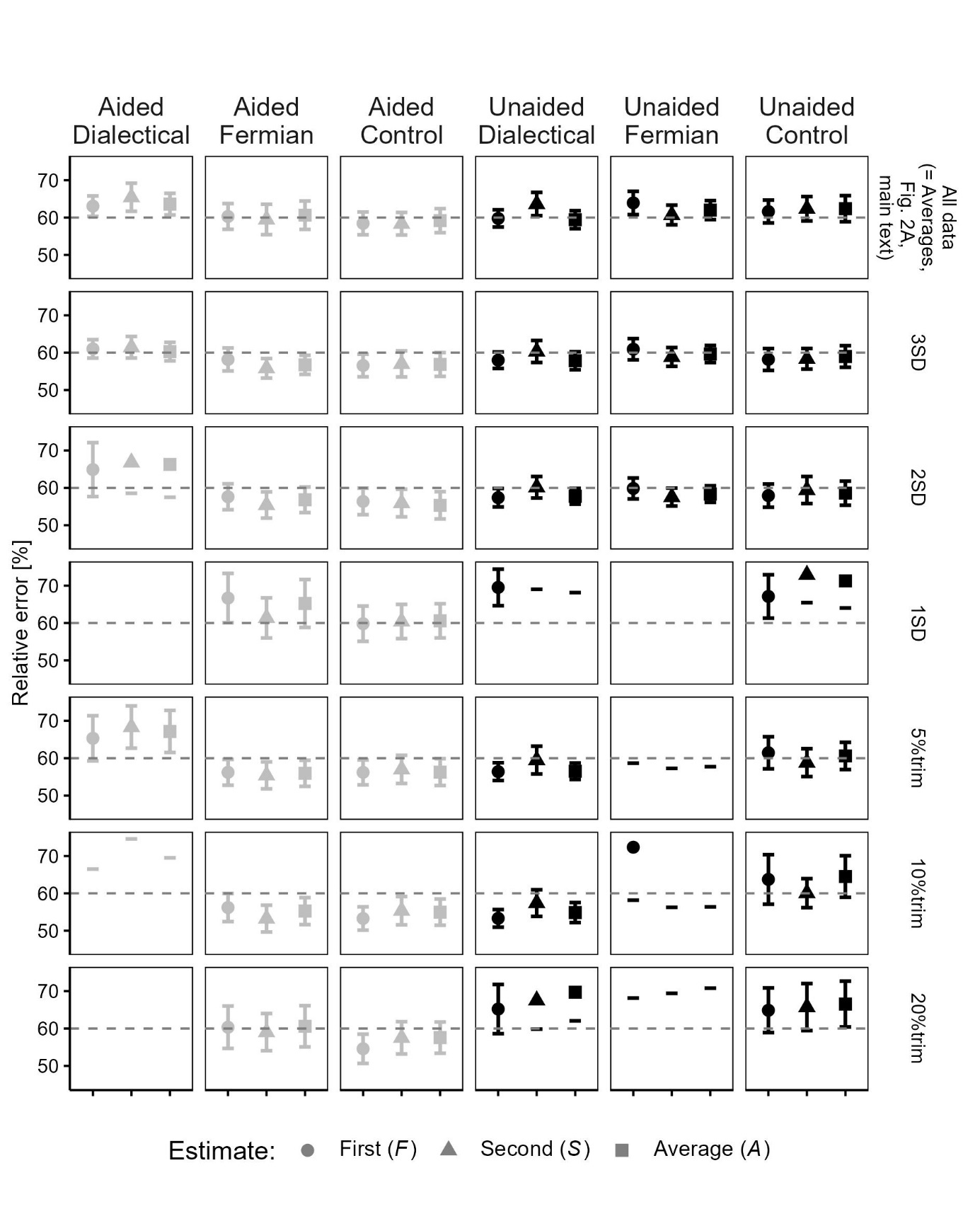


**Fig. S29.** Relative error of First (*F*) and Second (*S*) estimates, and their Average (*A*) when compared to the True value (*T*). All relative errors are averaged (within a given condition; Equation 2) across participant-specific medians (Equation 1). Vertical lines represent the +/- 1 standard error of these averages. The dotted horizontal lines aid comparisons of relative errors of *S* and *A* with the relative error of *F* within each condition. Comparisons of relative errors of *S* across conditions cannot be made because they had different reference points, namely the relative errors of *F* in those conditions, and these differences cannot be attributed to different treatments but only to sampling error. Some data points are not displayed because they exceeded the upper limit of the scale. The six panels in the top row display averages of the relative errors across all data and are identical to Fig. 2A in the main text. The panels in the six lines below display averages of participant-specific relative errors that were computed after excluding items based on various cutoff criteria, namely 3SD, 2SD, 1SD, 5%trim, 10%trim, and 20%trim.

## Distribution of error-reductions


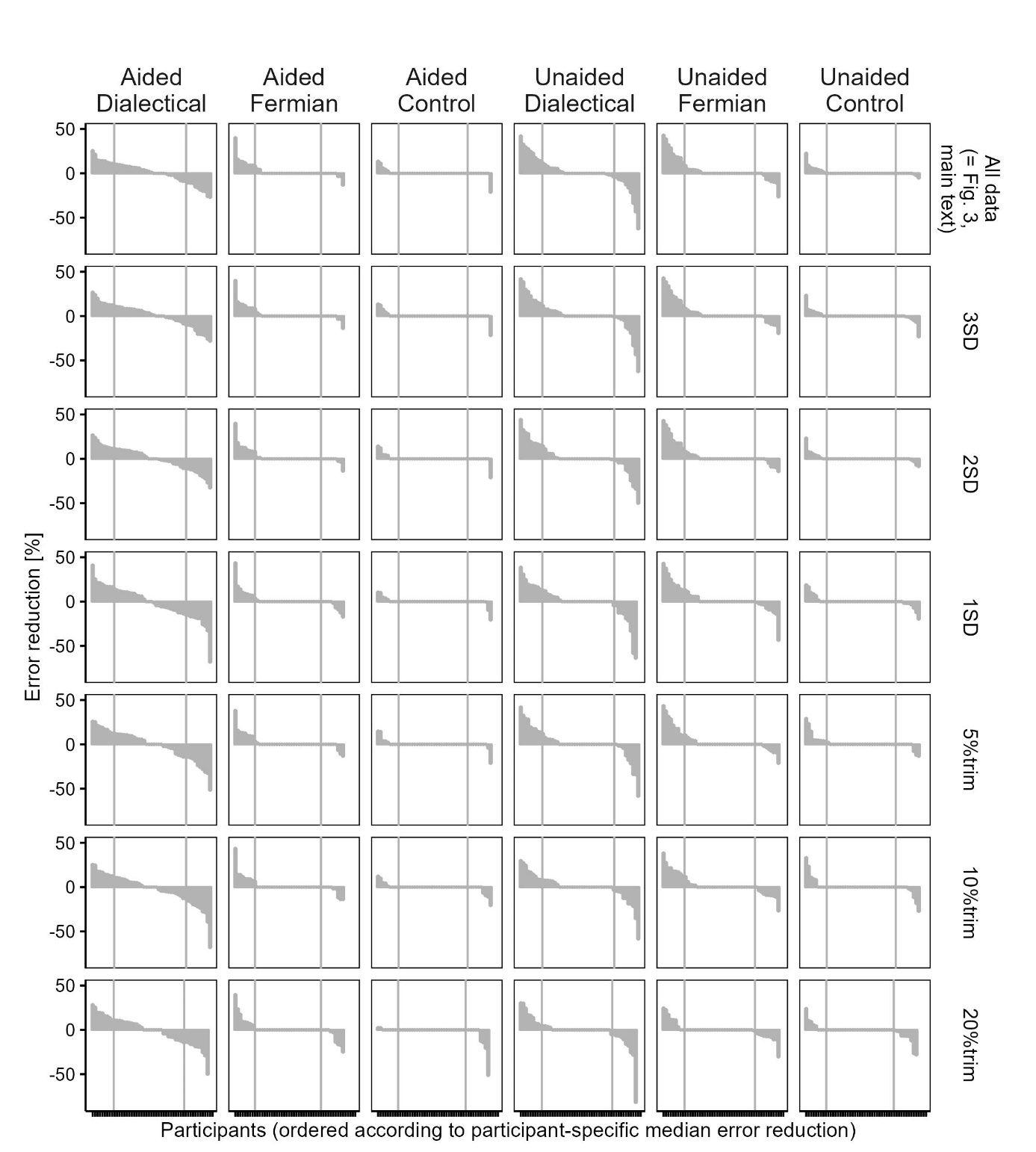


**Fig. S30.** Distribution of participant-specific median error-reduction of average estimates compared to first estimates (Equation 3), separately for the six experimental conditions.

The six panels in the top row display the distributions of participants’ median error-reductions that were computed using all data and are identical to Fig. 3 in the main text. The panels in the six lines below display the distributions that were computed after excluding items based on various cutoff criteria, namely 3SD, 2SD, 1SD, 5%trim, 10%trim, and 20%trim. The two vertical lines within each panel mark the 20 percentile and 80 percentile of each distribution. Even though these percentiles played no role in the trimming on the item level, we included them to illustrate that participant-trimming could potentially be performed after item-trimming.

## Average error-reduction


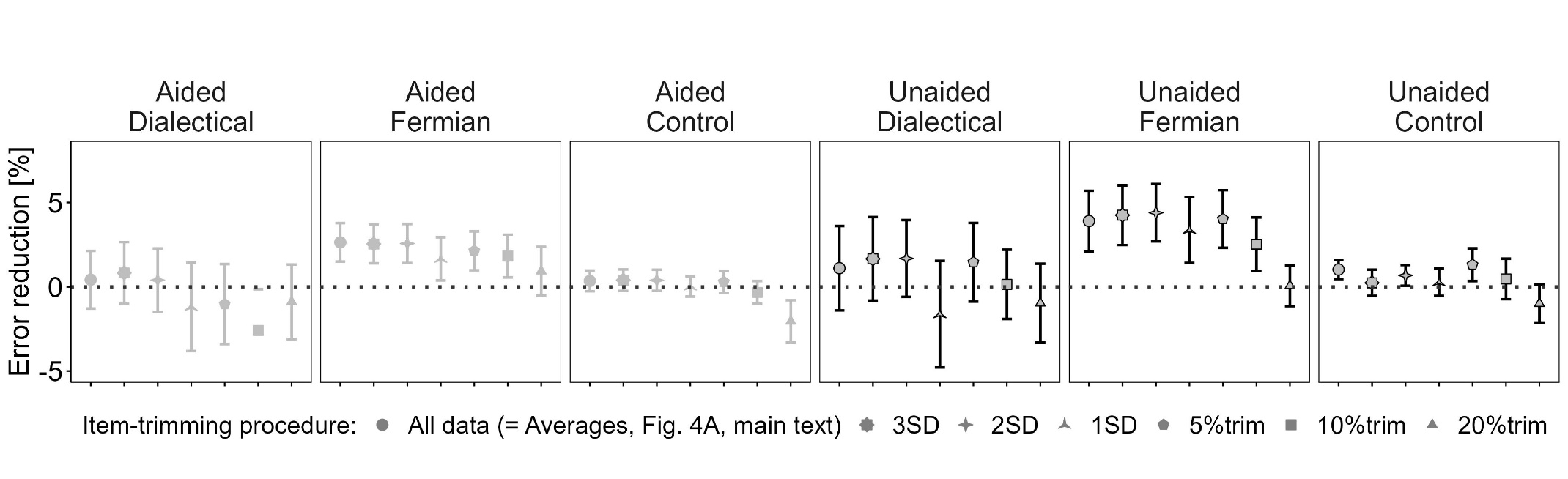
 **Fig. S31.** Averages (Equation 4) of participant-specific median error-reductions. Vertical lines represent the +/- 1 standard error of participants’ averages. The left-most entry in each panel displays the average median error-reduction across all data. These six left-most entries are floated in Fig. 4A in the main text. Whereas Fig. 4B in the main text displays participant-trimmed averages, the other entries in the present Fig. S31. display averages that were computed after excluding items based on various cutoff criteria, namely 3SD, 2SD, 1SD, 5%trim, 10%trim, and 20%trim.

## Commonalities and differences between item-trimming and participant-trimming

When comparing the effect of item-trimming reported above (not much of a difference between using trimmed and untrimmed data) to the effect of participant-trimming reported in the main text (substantial differences between using trimmed averages and untrimmed averages, in particular in the central Fig. 4), one is left wondering which of the two ways of implementing trimming is preferable. Item-trimming and participant-trimming share at least three commonalities and they differ in at least four ways. We start with the former.

Both ways of trimming proceed in three steps and precisely these three are their commonalities. In the first step, all data points—be it participant-specific metrics or item-specific measures—are rank-ordered. In the second step, cutoff points are determined, either relative to the distribution (e.g., the 20-percentile and the 80-percentile), or by some deviation around a central tendency (e.g., +/- 1 standard deviation).^[[1]](#footnote-2)^ These cutoff points split the distribution—be it of participants or of items—into three areas (left, middle, right) which can, in turn, be reduced to two areas (middle and tails). In a third step, the analysis or computation proceeds with only those data points—be it of participants or of items—located in the middle. In this third step, any data point in any of the two tails will no longer make a difference, that is, once the cutoff points are determined, it does not matter anymore whether it is located, say, 3 or 4 standard-deviations away from the average. Does trimming exclude outliers from the analysis? When considering step 1, the answer is no, and when considering step 3, the answer is yes, and both answers hold true both for item-trimming and participant-trimming. We now turn to four differences.

First, participant-trimming can essentially be conceived of as ‘sampling’ on the dependent variable of interest. Specifically, participant-trimming is mathematically equivalent to excluding 40% of the participants from the computation of the 20% trimmed average of the metric of interest (i.e., percentages of constellations in Fig. 1; accuracies of estimates in Fig. 2; participant-specific median error-reduction in Fig. 3 and condition-specific averages in Fig. 4) and that exclusion is determined based on participants’ value on that metric. In contrast, our item-trimming is not directly based on any dependent metric of interest, but on *F* and *S*, which, in turn, are used in Equations 1-4 to compute the aforementioned metrics.

Second, as a corollary of our first point, when using participant-trimming, the metrics of interest will, most likely, be computed from different participants for different metrics. In contrast, when using item-trimming, all metrics will be computed from the same data that remain after trimming.

Third, item-trimming permits targeting outliers at the level of the item, whereas participant-trimming only permits targeting outliers by excluding 40% of the participants (with *all* their data) from computing the 20%-trimmed average of the metric of interest. That said, if a participant from a given condition produced, for each item, either *F* or *S* (or both) that outlie relative to the *F* and *S*, respectively, of other participants of the same experimental condition and the same item, item-trimming would result in the exclusion of *F* and *S* of that item of that participant. In contrast, if only a few of that participant’s *F* and *S* were to be outlying, only these few items would be excluded but not the rest.

Fourth, participant-trimming and item-trimming differ in the assumptions those procedures make about the quality of data produced by participants in a study. Arguably, participants in a study may produce outlying data points. It seems reasonable to expect that some participants may produce a few responses that are inadequate for further processing, for instance, because those participants were occasionally distracted while hitting a key. It is even possible that a few participants may produce *only* inadequate data points, such as when a participant consistently clicks keys randomly, is a bot and not a human, or is under drugs. Whereas participant-trimming can be thought of as assuming that *all data* from about 40% of the participants are inadequate and all data from the *other* participants are adequate; item-trimming does not necessarily make that assumption. Rather item-trimming can be thought of as assuming that inadequate data will occur at the level of items, whilst being open to the possibility that it may also occur at the level of participants: namely when all items from a given participant are outlying, and hence inadequate for further processing. We believe that the probability that inadequate data is scattered across different participants is higher than the probability that only a subset of participants produced all of them.

Considering the four abovementioned differences, we posit that item-trimming is a more adequate procedure than participant-trimming. Still, we decided to report the former ‘only’ here in the Supplementary Information, and the latter in the main text because our main focus was to gauge the effect of the established participant-trimming procedure used by H&H (Goal 4, main text).

# Section E: Descriptive Analyses of Follow-up Questions

Table S2

Question 1: “Did you study biology…”

|  | **Aided Dialectical** | **Aided Fermi** | **Aided Control** | **Unaided Dialectical** | **Unaided Fermi** | **Unaided Control** |
| --- | --- | --- | --- | --- | --- | --- |
| Yes | 4 | 5 | 4 | 3 | 2 | 5 |
| No | 45 | 40 | 43 | 46 | 46 | 42 |

Table S3

Question 2: “Do you work with animals…”

|  | **Aided Dialectical** | **Aided Fermi** | **Aided Control** | **Unaided Dialectical** | **Unaided Fermi** | **Unaided Control** |
| --- | --- | --- | --- | --- | --- | --- |
| Yes | 1 | 2 | 1 | 0 | 0 | 3 |
| No | 48 | 43 | 46 | 49 | 48 | 44 |

Table S4

Question 3: “How good is your knowledge…”

|  | **Aided Dialectical** | **Aided Fermi** | **Aided Control** | **Unaided Dialectical** | **Unaided Fermi** | **Unaided Control** |
| --- | --- | --- | --- | --- | --- | --- |
| 1-not good at all | 3 | 1 | 2 | 4 | 2 | 1 |
| 2 | 13 | 15 | 11 | 9 | 16 | 7 |
| 3 | 13 | 10 | 10 | 16 | 14 | 16 |
| 4 | 12 | 11 | 14 | 18 | 12 | 15 |
| 5 | 7 | 5 | 10 | 1 | 4 | 7 |
| 6 | 1 | 2 | 0 | 1 | 0 | 1 |
| 7-very good | 0 | 1 | 0 | 0 | 0 | 0 |

Table S5

Question 4: “Can you describe one (or two) of the decision strategies…”

| **Condition**  **(n answers)** | **Participant ID** | **Description of the decision strategy** |
| --- | --- | --- |
| Aided | 22 | “Thought about their size and drew upon any knowledge I had from any nature documentaites” |
| Dialectical | 36 | “Visualised the animal and tried to imagine how the environment it comes from may impact its lifespan, sleep etc.” |
| (n=10) | 109 | “I tried to think if I had any knowledge of the particular animal and of patterns in different species. I made estimates based on small bits of knowledge or by comparing them with other animals. the ranges were influenced by how confident I was about my answer and what I thought the absolute minimum and maximum could be. Mostly guessing” |
|  | 110 | “My strategy will have failed because I am not too familiar with kg, prefer stones and pounds. As I do know even know my own weight or brain weight in Kg found it hard to estimate that of animals but really did try my best. perhaps just detailing the the average human weights at the start of the task would have helped. I just guessed an average adult weight of 65kg and tried to visualise the animal size to guess their weight. I admit brain weight was pure guesswork. Sorry. As i found the task so difficult I think my decision strategy probably failed” |
|  | 133 | “It was mostly just gut feelings to be honest and if I wasn't sure I would try to compare the animal to humans to try to come up with an estimate. If that wasn't an option then I would compare it to something I'm more familiar wit i.e. wolfs to dogs, tigers to cats etc.” |
|  | 180 | “I made decisions based on their size, where they fall in terms of predatory and poaching, how smart I believe them to be” |
|  | 190 | “I used the larger mammals as being the heaviest, living longer and having heavier brains, and dropped my estimations for the smaller mammals. But I found it very difficult to estimate at all.” |
|  | 230 | “I was estimating the lifespan of the animals that i knew most about , brain weight i knew nothing about . Fot the foxes i guessed weight on my dogs size and weight .” |
|  | 260 | “With regard to weight, I tried to compare the animals to an average size human of around 12 stone. Again I used human gestation of around 9 months to try to work out the gestation of the animals. The weight task was particularly time consuming and problematic as I don't work in kilos and had to spend a lot of time converting my answers from stone to kilo. Again I tried to use body size to brain weight those that was an area I probably got a lot wrong. It was a really hard study.” |
|  | 282 | “I was trying to compare to things I knew, like my own weight. For brain size i was imagining meat in a packet. Sleep time was mostly whether I thought they slept more or less then humans, which I put at 8 hours. Gestation period I also compared to humans, but I couldn't remember if our gestation was considered short or long, so that didn't help a lot. I have good knowledge of animals generally but not these things apparently. I used the fact that intelligence is linked to brain size when estimating. I also believe that intelligence correlates with sleep length so I used that where possible. I tried to imagine how developed babies are at birth from the species in question, as I know we are born more premature than other species, but I also assumed we took longer to develop than most so that was a little confusing to estimate.” |
| Unaided Dialectical | 12 | “I found this really difficult! I guess I just tried to think of the species as a whole and what kind of conditions they live in in the wild” |
| (n=10) | 57 | “Honestly, most of my decisions were based around my knowledge of humans and how similar I thought certain animals were to humans. So for example, sleep time I based around an average of 8 hours, although I slightly changed that for the wolves and the tigers as I know cats and dogs sleep for longer. However, gestation periods and life span really made me wander and I was very unsure. I am also notoriously bad at estimation as I struggle to picture what numbers mean in real life so, with weight I tried to think of bags of sugar (one kilogram being one bag of sugar) to help me visualise it. This helped I think with the brain estimations but not with the body weight estimations.” |
|  | 79 | “I tried to equate where possible with my limited experience of domestic animals (e.g. dog/wolf)” |
|  | 94 | “trying to remember things i'd heard in documentaries and knowledge” |
|  | 171 | “I was comparing it to humans.” |
|  | 186 | “I looked to see if the animal animal was a predator or a food source, from this you could guess the gestation period and brain size and mass.” |
|  | 202 | “I have spent a lot of time at Zoos and have a strong interest in animals so I based my existing knowledge on this and similar animals characteristics i.e. similarities between zebra and horse.” |
|  | 229 | “I used what I knew about similar species. For example, when answering about the gestation period and life expectancy of the Bengal tiger - I thought about what I knew about domesticated cats. I also had in my mind the average weight of a human, and considered whether I thought the animals were heavier or lighter. I also tried to picture these animals from seeing them in person at the zoo. This strategy could perhaps fail if these animals have different outcomes in the wild - shorter average life expectancy due to predators, lower average weight due to reduced access to food etc. “ |
|  | 246 | “Using my body weight for a baseline- harder to guess for something 6 times your size! Using the hunt and rest patterns to help gage sleeping patterns- but I don't know enough about wild animals to make a completely educated guess.” |
|  | 268 | “i pictured the animal in my mind and how i thought they would live in the wild,smaller animals i thought would live a shorter live due to being hunted by predators,the weight of the brain i thought about the actual body weight and then estimate the brain size from that.” |
| Aided Fermi  (n=10) | 18 | “i used the only knowledge of the animal i had which is their size relative to each other, bigger animals generally have longer gestation periods and probably bigger brain weight.” |
|  | 43 | “Tried to recall previous knowledge of the species or a similar animal and used that as a starting point. This would only work well if the animals were closely related but even then isn't fail-safe.” |
|  | 80 | “I considered what I thought I knew about similar animals.” |
|  | 112 | “It was almost pure guesswork. I went by size of animal and inferred they would usually have smaller brains, quicker gestation and shorter lifespan” |
|  | 141 | “I tried to base it on my general knowledge form seeing soem of the animals in the wild, or at zoos, or I suppose mostly from learning about them from natural history / animal documentaries on TV. I tried to give my best estimate and also made comparisons to our own dog's weight, and ny own weight.” |
|  | 197 | “I really have no idea, I just had to guess most of them as we were not allowed to look things up. One or two I happened to have a clue about, for instance, I am fairly certain the gestation period of a horse is 11 months and that of an elephant, 13. Other than that, I could only consider what little I know about any animals and try to apply that. I know domestic cats sleep a lot so it might follow that a tiger would as well. I considered how much things are hunted, either by natural predators or by man as that could affect their life span. I really don't know what you want me to say.” |
|  | 218 | “All I can go on is nature documentaries. To be honest though I really don't have a great brain for statistics and storing facts. I know a lot about animals that I have been interested in, but not much about sleep cycles and gestation times.” |
|  | 237 | “I thought of animals of a similar size build or species and made a comparison based on that. So my strategy in the initial part of the study was similar to that we were instructed to use in the second part of the study. I might for example have compared my estimations for a zebra with my estimations for a horse. I think it would fail where looks are deceptive and the similarities/differences between a species are based more on other factors than appearance.” |
|  | 278 | “I tried to think of animals I know a little about. I also compared them to my knowledge of human biology. This would be most accurate where the animals I thought about were the same species, but not as accurate for animals I am unfamiliar with.” |
|  | 290 | “I tried to create a clear image of the said animal in my mind, I tried to visualize their overall body size and strength and tried to use common sense to relate that to their brain size i.e. a larger animal is more likely to have a larger brain. Additionally I tried to think about for example the animals in the wild, they would need to be more alert of any prey/predators and therefore maybe have less sleep than animals not in the wild.” |
| Unaided Fermi | 31 | “I know how much I weigh and how much my toddler weighs, so I am guessing with those weights as a comparison to sizes. I also was guessing brain size in relation to the size and weight of the animal, the bigger the animal, the heavier the brain.” |
| (n=9) | 55 | “I made weight assumptions loosely based on my perception of how large the animals were. This was more difficult for larger animals, as I have little first hand experience of seeing or being near these types of animals. I also have an assumption that brains found in animals are not particularly large/heavy, but have no idea where this assumption arises from. Once I had made a decision on a ballpark figure for a type of animal, I used that as a reference for decisions on other animals. This strategy could be completely misguided if the initial estimate was way off.” |
|  | 106 | “I was just trying to base it on the animals weight relative to mine, whether or not I was successful is another matter. The dolphin I didn't have a clue about, was it like a flipper dolphin or one of those little ones you get? And I've never really thought about wild animals lifespans so I could also be off by a lot there. And now that I've thought about it a bit more I definitely overestimated how big pandas are.” |
|  | 115 | “I worked out my own body weight in kg and imagined 'how many of me' would a certain animal be. I think with my estimations in section1, were not wide enough on reflection, I should have put a larger scale range to cover both extremes. My technique of visualising myself was easier for animals of a similar size or larger (e.g. x2 of me) however was very hard to work out smaller animals.” |
|  | 143 | “I was using my logic and common knowledge.” |
|  | 172 | “I thought of similar animals that I could estimate better and compared them (similar to strategy asked on block 2). Sometimes I had to fall back on humans!” |
|  | 173 | “I tried to compare them to dogs, which is the only animal I really know much about” |
|  | 192 | “in the first part i tried to think of similar animals or remember from facts ive learned in the past.” |
|  | 249 | “i tried to remember when id read articles or news about animals” |
| Aided Control  (n=9) | 14 | “I roughly compared to humans and made best guesses against any existing knowledge, I knew elephants lived long and spent a long time pregnant but guessed for smaller animals like goats, horses etc. I went with the little knowledge I have and then made a rough guess of how well I knew. For instance, I think I knew the rough weight of human brains and tried to scale this down.” |
|  | 45 | “Some decisions I felt more confident on as the range 'should' be smaller - gestation. Worked on seasonal cycles and food abundance, plus state of young on birth etc. I realised that I was really unsure about brain weight (as i did not know if to factor in water!), but did this influenced by life strategies/body weight. Life span - based on as much as I could remember about life histories of the species! Was unsure to include infant mortality, so all lower estimates set at 6 months. Upper was greatest/average was as I could imagine based on life histories. Body weight - based on size, muscle mass, life strategies - compared to my own size and body weight. I realised that the total range I should use for estimation was unsure. Sleep time - based on habitat, predator/prey/ diet, communal living, need for vigalence etc.” |
|  | 52 | “Generally larger animals with a less risky lifestyle probably live longer; slow or sedentary animals with fewer natural predators sleep longer; gestation periods I roughly compared to body size with a human 9 month gestation as a baseline (thinking that humans have a relatively long gestation as our babies have well developed brains), and also the amount of 'resource' an animal needs to absorb from its environment to reproduce vs the risk of a long gestation. Sleep periods: if an animal seems a nervous, alert sort of creature in a potentially hostile environment, I guessed it slept in shorter bursts than something big and sluggish like a panda. Quite a lot of that was guesswork, esp. the weights!” |
|  | 83 | “I used some knowledge - I know that, for example, primates are more like humans, and that "big cats" will be similar to "small cats" - goats are a bit like sheep. and wolves/foxes are a bit like dogs. In all honesty some of it was guesswork, and I'm happy to admit it.” |
|  | 161 | “In estimating I compared each animals characteristics to myself and how they would live in the wild. Not all other species are the same as humans so this strategy has flaws.” |
|  | 181 | “I felt that the biggest animals had the longest gestation periods.. Not having much experience with animals i did struggle a bit with this task but gave it my best efforts” |
|  | 238 | “gave serious thought to answer detail based on whatever knowledge i have then tried to keep range estimates as sensible as possible extending the upper range further when really doubtful of answer” |
|  | 250 | “I tried to picture the animal and its size compared to me. I am about 65kg and tried to estimate weight from that. The life span and gestation was a guess from what i remember of the wildlife documentaries i have watched in the past. The brain size was just a guess depending on wha i thought the overall body weight was and how clever i think the animals are.” |
|  | 252 | “I used bodyweight to decide sleep time, due to how much energy the animal would need to carry its weight. For gestation, I also considered the size of the animal and intelligence of the animal. A lot of decision strategies were based on size and intelligence of the animal.” |
| Unaided Control | 21 | “I tried to think of how many natural predators the species had as if they are constantly hunted, that would reduce their average lifespan. I also tried to think of their environments, a goat may come across less danger than a tiger.” |
| (n=9) | 35 | “My estimations were based in large part upon guesses as to the overall body weight of the animal in question - I am not entirely sure, however, whether this strategy works at all and will be interested to see how poor my guesses were!” |
|  | 58 | “tried to imagine each animal and then in comparison to previous animals in regards to general size” |
|  | 156 | “My maxima and minima in the first block were in two narrow a range, consequently I widened these in block 2. Clearly the second strategy is more likely to be successful I think.” |
|  | 178 | “The size of the animal and comparing it a human and averaging it out that way” |
|  | 207 | “there are certain animals that i was more familiar with than others so i had a better idea on some of the answers i made, i also took into account the type of animal ie wolf which is dog like and dogs usually live to around a certain age and i tried to compare that with the chimp which belongs to the monkey family” |
|  | 222 | “I tried to work out how heavy they would be by comparing the size of them to my body weight and estimating the weight. I tried to estimate the lifespan by the amount of predators they had and what climate they lived in. Brain size was based on the animal. I know that even though an animal is huge it doesn't have to have a huge brain.” |
|  | 245 | “I tried to compare a known weight e.g. a bag of sugar for 1kg or my own weight of 68kg, and think about if it would be less or more than that and by how much. I also thought that the heavier and more humanlike the animal, the longer they would live.” |
|  | 280 | “I just tried to use my general knowledge about animals, it was a lot of guess work, if I was really unsure I would try to use other similar animals that I know of and estimate on my knowledge of them to help me with my answers, some of which I did second guess myself.” |

*Note.* Participants’ texts were not edited.

Table S6

Question 5: “Can we trust your data…”

|  | **Aided Dialectical** | **Aided Fermi** | **Aided Control** | **Unaided Dialectical** | **Unaided Fermi** | **Unaided Control** |
| --- | --- | --- | --- | --- | --- | --- |
| Yes* | 49 | 45 | 47 | 49 | 48 | 47 |
| No** | 0 | 2 | 2 | 0 | 1 | 2 |

*Notes.*

* The raw data of these 285 participants can be accessed on OSF page (https://osf.io/kwdfg/?view_only=c3b7a7ada7804768a9b8dcff71ab0311) folder “Trust data”, the name of the file: data_WOW_27.march.23.Trust.csv.

** The raw data of these 7 participants can be accessed on OSF page (https://osf.io/kwdfg/?view_only=c3b7a7ada7804768a9b8dcff71ab0311) folder “No trust data”, the name of the file: data_WOW_27.march.23.NoTrust.csv. As we explained in the Method section of the manuscript (subsection Participants), we excluded these 7 participants from the analysis. Consequently, the answers of these 7 participants to the follow-up questions of Part 4 are also not included in the descriptive statistics of the present Section D.

Table S7

Question 6: “Did you seek any outside help….”

|  | **Aided Dialectical** | **Aided Fermi** | **Aided Control** | **Unaided Dialectical** | **Unaided Fermi** | **Unaided Control** |
| --- | --- | --- | --- | --- | --- | --- |
| Yes | 2 | 2 | 3 | 4 | 3 | 5 |
| No | 47 | 43 | 44 | 45 | 45 | 42 |

Table S8

Responses to the second part of Question 6: “If yes, please specify how many Estimation Questions you sought help with. *Please answer honestly; your payment will not be affected by your answer.”

| **Condition** | **Participant ID** | **Answers of participants who answered “Yes” to Question 6** |
| --- | --- | --- |
| Aided Dialectical (n=1) | 190 | “I sought help with the first couple of questions because I had no idea where to start my estimations for weight. Once I had a rough idea of animal weights I estimated the remaining. For gestations and sleep time I completely estimated.” |
| Unaided Dialectical (n=1) | 246 | “for the elephant, dolphin and zebra” |

*Note.* Participants’ texts were not edited.

Table S9

Question 7: “Did you use the fullscreen view…”

|  | **Aided Dialectical** | **Aided Fermi** | **Aided Control** | **Unaided Dialectical** | **Unaided Fermi** | **Unaided Control** |
| --- | --- | --- | --- | --- | --- | --- |
| Yes | 48 | 44 | 44 | 46 | 46 | 46 |
| No | 1 | 1 | 3 | 3 | 2 | 1 |

Table S10

Question 8: “Do you have any comments…”

| **Condition**  **(n answers)** | **Participant ID** | **Final comments** |
| --- | --- | --- |
| Aided | 109 | “no” |
| Dialectical (n=8) | 110 | “I did have to read the comparison question a few times to understand it but the rest of the instructions were very clear. As mentioned above indicating the average adult details in kg before the task would have helped me personally as I deal with imperial weights” |
|  | 133 | “I wasn't sure if the lifespans should take into account if the animal is prey but I assumed this would be the case.” |
|  | 188 | “Everything was fine, quite a fun study, Would like to see how my estimates fall alongside the reality” |
|  | 190 | “Instructions were clear and the study was interesting but I'm not sure how well I did.” |
|  | 230 | “Very interesting , really enjoyed this study and it makes me wonder about finding out more about the animals i knew least about .” |
|  | 260 | “Did not enjoy at all!!! It would have cut down a lot of time if you had also had imperial weights.” |
|  | 282 | “All clear, no issues. Generally enjoyable, did get tedious towards the end.” |
| Unaided Dialectical | 57 | “I really struggled to understand what the different breeds of animals were, so I know an elephant but don't know the difference between different elephants, the same goes for most of the animals.” |
| (n=8) | 94 | “no” |
|  | 171 | “All very clear” |
|  | 186 | “no” |
|  | 202 | “No.” |
|  | 229 | “The instructions were clear. It would be useful to have had the weight measurements available in stones and pounds - living in the UK, I'm used to measuring my own weight in stones and pounds (not kilograms and grams). I had to picture a 25kg sack of potatoes as a reference.” |
|  | 246 | “Instructions were clear, questions made sense. Maybe place the animals in order, e.g. all of the tiger questions together etc to help make it easier to recall in the second stage.” |
|  | 268 | “the instructions were clear,but i think it would of been easier to do the second estimates if we were givern the first estimates to work from” |
| Aided | 18 | “no” |
| Fermi | 43 | “No” |
| (n=10) | 80 | “No.” |
|  | 112 | “no” |
|  | 141 | “A very interesting study to do, and quite difficult to make the estinates - i hope my entries prove relevant!” |
|  | 197 | “I don't understand why you had to have tonnes in the equation, very confusing since most animals weigh nothing like that much. Having to convert everything to kgs when I still think in pounds was a pain. No, I did not enjoy this study since I had no clue about many of the answers and no-one enjoys being made to feel stupid.” |
|  | 218 | “I hope I did this correctly.” |
|  | 237 | “The instructions were clear and easy to follow.” |
|  | 278 | “Interesting study...It made me realise how little I really know about animals!” |
|  | 290 | “Was hard at times but fun and made me challenge myself. Everything was clear. Thank you.” |
| Unaided Fermi | 31 | “The instructions were clear andeasy to understand. The questions made sense. The study was interesting . There were no mistakes that I noticed.” |
| (n=9) | 55 | “The instructions were clear and the study was more enjoyable than I originally anticipated. I think it has made me aware that I have a poor overall perception of weight.” |
|  | 106 | “Can't think of any. Good luck with your research.” |
|  | 115 | “Very interesting, think I got better as I went along. Although, my animal knowledge was poor it was good fun. Instructions were clear.” |
|  | 143 | “It was very demanding and tiring.” |
|  | 172 | “Would be interested to know what you were researching” |
|  | 173 | “No” |
|  | 192 | “no” |
|  | 149 | “no” |
| Aided Control | 45 | “I need to brush up on some stuff! Bit gutted I struggled with brain weight! Thanks, really enjoyed that. Yes, made sense - would be good to clarify life span - should this include infant mortality? Brain weight - with or without water?” |
| (n=9) | 52 | “That was fun, but I could have done with a reference point for weights (how much does a human weigh in kg? How heavy is a human brain?). But that might have affected the answers I gave.” |
|  | 83 | “The instructions were clear, unfortunately, being unfamiliar with many wild animals, I guessed and gave a large confidence interval.” |
|  | 123 | “none” |
|  | 161 | “Interesting study.” |
|  | 181 | “The task was easy to follow with clear instructions” |
|  | 238 | “clear instructions - but very demanding to maintain concentration - particulaly in the second set” |
|  | 250 | “I found the study interesting. I found the instructions clear and easy to understand.” |
|  | 252 | “None” |
| Unaided | 58 | “no” |
| Control | 156 | “No” |
| (n=6) | 178 | “Very interesting study would be keen to see the answers” |
|  | 207 | “thankyou i enjoyed taking part in this study a lot” |
|  | 245 | “None, I enjoyed it even though I think I got a lot of answers very wrong.” |
|  | 280 | “instructions were clear and I really enjoyed the study, I have had to dig deep into my brain to think about my knowledge of animals, which may be good or maybe bad.” |

*Note.* Participants’ texts were not edited.

# Section F: Additional Discussion of the Strategies

The consider-the-opposite strategy and the Fermian strategies can be compared with regard to two criteria, which can be captured by the following two questions. First, to what extent do these strategies boost the wisdom-of-the-inner-crowd? In other words, what is closer to the true value: *F* or the average of *F* and *S*? Note that this was the central question and hence the focus of our research; it was prompted by HH09 that served as our starting point. Second, how accurate are the estimates generated by these two strategies? Note that we also reported results speaking to this question, namely, in Figure 2 where we reported the relative error of *S*. Recall that only participants’ second estimates, *S*, were generated after having been instructed to use one of the two strategies, consider-the-opposite or Fermian, while all their *F* have been generated in a naïve way, that is, without any instruction on how to do this.

Our result section focused on the first question (To what extent do these strategies boost the wisdom-of-the-inner-crowd?), with the relative error reduction (which measures what is gained from using the average, *A*, instead of *F*) at its center. In our discussion section, we broadened the perspective, thereby also addressing the second question (How accurate are the estimates generated by these two strategies?).

The difference between these two questions is marked by the importance of the first estimate *F*, which, in turn, marks an important difference between the two strategies. As we said in §6: Fermian guesstimation does not build on any *F* that has been generated before. When we, in our experiment, nevertheless asked participants to generate a naïve *F* before we asked them to use a similarity-based (i.e., Fermian) strategy to generate *S*, we simply did this in order to follow the logic of the wisdom-of-the-inner-crowd-paradigm that requires to average *F* and *S*. It would, however, also have been possible to instruct participants to use such a Fermian strategy right from the start, that is, when generating *F.*^[[2]](#footnote-3)^

Relaxing the embeddedness of the strategies within the wisdom-of-the-inner-crowd-paradigm opens avenues for new research. Recall that the wisdom-of-the-inner-crowd-paradigm implies averaging *F* and *S*, and recall that it is the experimenter who performs this averaging. Alternatively, one could let participants integrate the wisdom of their inner crowd (see also Herzog & Hertwig, 2014). For dialectical bootstrapping this could be done as follows: Participants are asked to (1) generate their best estimate, and to (2) write it down. Right afterwards, they are (3) instructed to consider the opposite, (4) write down the estimate resulting from this exercise, (5) draw the conclusion themselves, and (6) write down their final estimate that integrates both estimates. For Fermian guesstimation participants are asked to use a similarity-based strategy. This could be done as follows: Participants are instructed to (1) write down the estimates of similar objects, (2) draw the conclusion themselves (i.e., integrate all estimates of similar objects), and (3) write down their estimate of the target object.

In other words, in future studies, one could compare (a) participants’ own integration of their own naïve *F* and the estimates they generate after having been instructed of considering the opposite, and (b) participants’ own integration of estimates of similar objects after having been instructed to use a similarity-based strategy to estimate a target object.

Note that this formulation mirrors a formulation we used in the discussion, where we suggested yet another idea for a future study: “the Fermian-strategies’ potential could be assessed further by instructing participants to produce Fermian estimates already for *F* (§6), allowing to test those Fermian *F* against the conditions we realized: (a) averages of naïve *F* and dialectical-bootstrappers’ *S*, and (b) averages of naïve *F* and Fermian *S*.”

These two ideas for future studies could be combined in a two-by-two design, with one factor Strategy (consider-the-opposite vs. Fermi) and the other factor Aggregation (averaging done by the experimenter vs. letting participants integrate). A third factor could be added by instructing participants, in one condition, to write down the elements they are then integrating themselves, vs. letting them, in another condition, do this integration in their mind without leaving written traces on their way (as implemented by Herzog & Hertwig, 2014).

# References

Qualtrics. (2021). *Qualtrics* (Version XM) [Computer software]. <https://www.qualtrics.com>

Herzog, S. M. & Hertwig, R. Think twice and then: Combining or choosing in dialectical bootstrapping? Journal of Experimental Psychology: Learning, Memory, and Cognition **40**, 218–232 (2014b).

Wilcox, R.R. & Keselman, H.J. Modern robust data analysis methods: Measures of central tendency. *Psychological Methods* **8**, 254–274 (2003).

1. We hasten to add that when Wilcox and Keselman (2003) discuss trimming as a procedure to obtain robust measures, they focus on percentiles. Note that percentiles as cutoff points offer more flexibility to classify outliers as they can, in contrast to standard deviations, have different distances to the central tendency of a distribution and are hence better suited for asymmetric distributions. Our decision to also use standard deviations when implementing item-trimming was purely driven by the desire to compare methods in an explorative manner. [↑](#footnote-ref-2)
2. When doing exactly this, one may want, to be on the safe side, to prevent participants from coming up with a naïve *F* by explicitly instructing them to *not* start with an estimate of the target object, but to start with estimates of similar objects. [↑](#footnote-ref-3)
